# Spatial–temporal heterogeneity and determinants of HIV prevalence in the Mano River Union countries

**DOI:** 10.1186/s40249-022-01036-1

**Published:** 2022-11-29

**Authors:** Idrissa Laybohr Kamara, Liang Wang, Yaxin Guo, Shuting Huo, Yuanyuan Guo, Chengdong Xu, Yilan Liao, William J. Liu, Wei Ma, George F. Gao

**Affiliations:** 1 Department of Epidemiology, School of Public Health, Cheeloo College of Medicine, Shandong University, Jinan, 250012 China; 2 NHC Key Laboratory of Biosafety, National Institute for Viral Disease Control and Prevention, Chinese Center for Disease Control and Prevention, Beijing, 102206 China; 3 CAS Key Laboratory of Pathogen Microbiology and Immunology, Institute of Microbiology, Center for Influenza Research and Early-Warning (CASCIRE), CAS-TWAS Center of Excellence for Emerging Infectious Diseases (CEEID), Chinese Academy of Sciences, Beijing, 100101 China; 4 State Key Laboratory of Resources and Environmental Information System, Institute of Geographic Sciences and Natural Resources Research, Chinese Academy of Sciences, Beijing, 100101 China

**Keywords:** Spatial distribution of HIV prevalence, Geodetector, Spatial stratified heterogeneity, Least Absolute Shrinkage and Selection Operator, Comprehensive correct knowledge, Machine learning, Africa

## Abstract

**Background:**

Utilizing population-based survey data in epidemiological research with a spatial perspective can integrate valuable context into the dynamics of HIV prevalence in West Africa. However, the situation in the Mano River Union (MRU) countries is largely unknown. This research aims to perform an ecological study to determine the HIV prevalence patterns in MRU.

**Methods:**

We analyzed Demographic and Health Survey (DHS) and AIDS Indicator Survey (AIS) data on HIV prevalence in MRU from 2005 to 2020. We examined the country-specific, regional-specific and sex-specific ratios of respondents to profile the spatial–temporal heterogeneity of HIV prevalence and determine HIV hot spots. We employed Geodetector to measure the spatial stratified heterogeneity (SSH) of HIV prevalence for adult women and men. We assessed the comprehensive correct knowledge (CCK) about HIV/AIDS and HIV testing uptake by employing the Least Absolute Shrinkage and Selection Operator (LASSO) regression to predict which combinations of CCKs can scale up the ratio of HIV testing uptake with sex-specific needs.

**Results:**

In our analysis, we leveraged data for 158,408 respondents from 11 surveys in the MRU. From 2005–2015, Cote d'Ivoire was the hot spot for HIV prevalence with a Gi_Bin score of 3, *Z-Score* 8.0–10.1 and *P* < 0.001. From 2016 to 2020, Guinea and Sierra Leone were hot spots for HIV prevalence with a Gi_Bin score of 2, *Z-Score* of 3.17 and *P* < 0.01. The SSH confirmed the significant differences in HIV prevalence at the national level strata, with a higher level for Cote d'Ivoire compared to other countries in both sexes with q-values of 0.61 and 0.40, respectively. Our LASSO model predicted different combinations of CCKs with sex-specific needs to improve HIV testing uptake.

**Conclusions:**

The spatial distribution of HIV prevalence in the MRU is skewed and the CCK about HIV/AIDS and HIV testing uptake are far below the threshold target set by UNAIDS for ending the epidemic in the sub-region. Geodetector detected statistically significant SSH within and between countries in the MRU. Our LASSO model predicted that different emphases should be implemented when popularizing the CCK about HIV/AIDS for adult women and men.

**Supplementary Information:**

The online version contains supplementary material available at 10.1186/s40249-022-01036-1.

## Background

After 40 years of hard work and global cooperation in the fight against AIDS, yet, in 2020, the world recorded 37.7 million people living with HIV including 10.2 million who were not in treatment, and 680,000 deaths from AIDS-related illnesses [1, 2]. Among those not on treatments, approximately 4.1 million did not know their HIV status. On top of this, new HIV infections remain unacceptably high at 1.5 million people despite tremendous efforts by world leaders for reducing AIDS-related death and new HIV infections to less than 500,000 by the end of 2020 [3, 4]. In the African region, 880,000 people acquired HIV, and 460,000 HIV-related deaths occurred in 2020 [4]. Specifically, in the Mano River Union (MRU), there were 6200, 5300, 1400 and 5400 new HIV infections in Cote d’Ivoire, Guinea, Liberia and Sierra Leone respectively in 2020, and the sub-region has one of the lowest comprehensive correct knowledge (CCK) about AIDS and HIV testing uptakes in Africa [5, 6]. The United Nations General Assembly agreed to end the AIDS epidemic by 2030 and assumed that interim targets (90-90-90), should be achieved by the end of 2020. However, the HIV epidemic has slipped such interim assumptions [6]. In 2020, new cases from countries in Western and Central Africa accounted for 37% of new HIV infections worldwide, leading the region far off the track of the 90-90-90 target for ending the epidemic [4].

Narrowing down our fight against HIV to a community-centred approach in low-income countries is vital to adequately leverage the available scarce resources as international funding declines [7, 8]. Numerous studies have indicated the potential benefits of adjusting HIV programs to focus on the populations and locations with the highest need for interventions [8, 9]. The broad array of CCK about HIV/AIDS in the Demographic and Health Survey (DHS) questionnaire(s) is so elaborate that it is very difficult for people with lower education to be able to remember them all, especially for those in post-conflict and fragile nations. These challenges are further compounded by the low literacy rate and a limited healthcare system that makes the situation even worse to improve the HIV testing uptake in these countries. CCK about HIV/AIDS is directly correlated with HIV testing uptake, and research has proved that adults with higher CCK have a higher ratio of HIV testing uptake than those with lower CCK [10, 11]. However, not all knowledge about HIV is relevant to every community due to varied demographic patterns and social dynamics, characterized by dramatic differences in socioeconomic status. Besides, due to differences in human cognition, different people might pay attention to different CCK items. Consequently, when disseminating CCK to different groups of people, it is necessary to have different focuses to ensure that the dissemination of CCK will significantly increase the rate of voluntary HIV tests.

Geographic Information System (GIS) tools are global headlines in recent epidemiological research [8, 12]. Looking at several types of indicators from a spatial perspective can integrate valuable context to human activities with the outstanding visualization benefits that maps can provide [8, 13]. For example, a dataset of health indicators containing their locations and attributes can decipher the patterns of population dynamics and then elucidate the implication of interaction between human activities and environmental factors which can lead to an increase in disease prevalence [8, 14, 15]. Linked DHS and Global Positioning System data are being used to improve the planning for familial interventions, profile the correlation between malaria prevalence and anaemia in children in West Africa, and analyze the effect of environmental factors accounting for early child mortality [16]. The Global Fund’s 2017–2022 strategy notes that fine, spatial and lower level estimates are essential for good decision-making and are a prerequisite for the success and long-term impact of HIV and other health programs [17, 18].

Spatial stratified heterogeneity (SSH) is a phenomenon that describes the differences within and between strata in geographic space. Geodetector is a novel statistical tool that measures SSH and its attributes among data [19]. Geodetector could also utilize q- statistics to test the interaction outcomes between one or more independent factors.

**Fig. 1:**
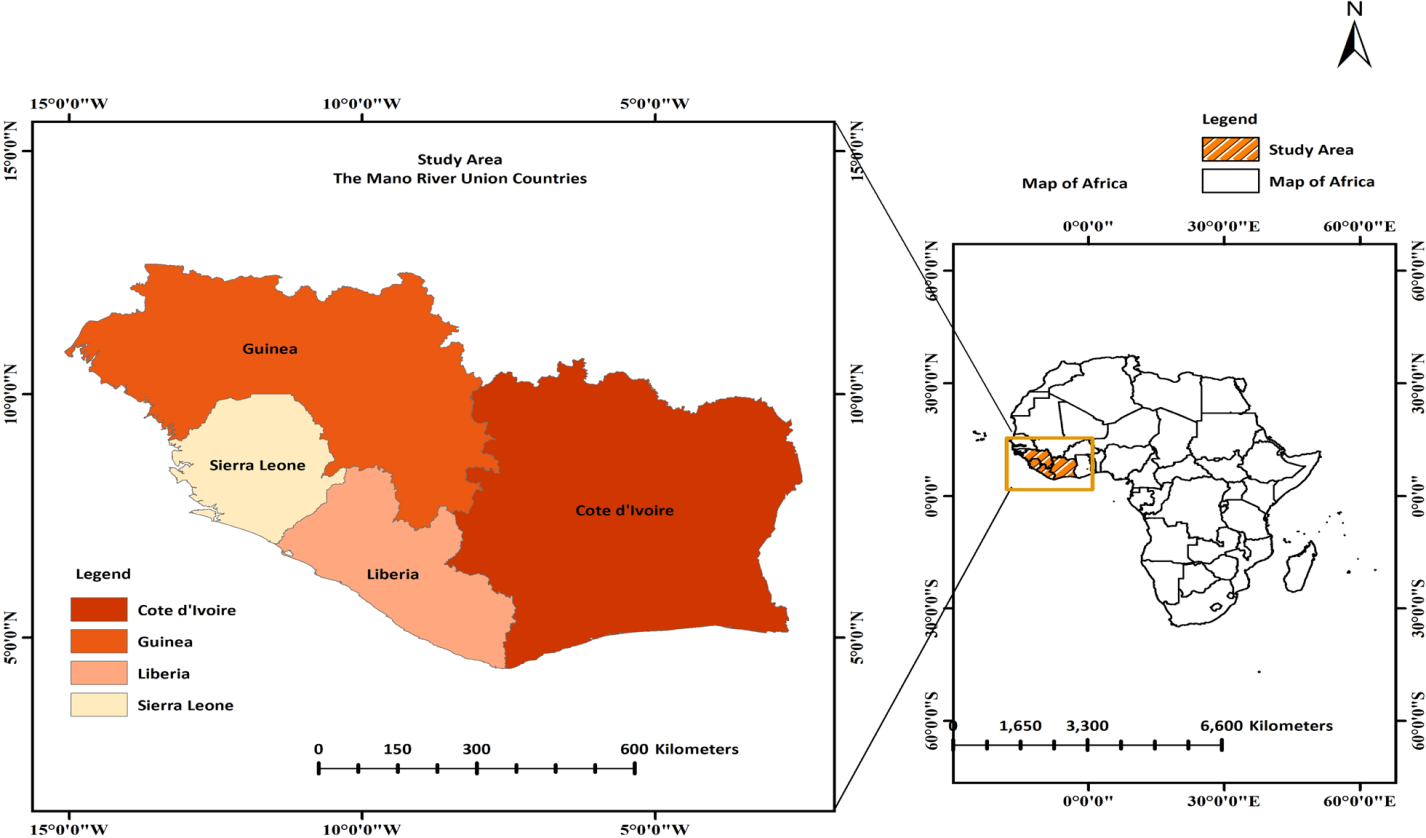
The geographic locations of countries that constitute the Mano River Union

The MRU is a sub-regional community with four countries (Cote d’Ivoire, Guinea, Liberia and Sierra Leone) located on the west coast of Africa with English and French as official languages [20, 21]. While Cote d’Ivoire and Guinea have French as their official language, neighbouring Liberia and Sierra Leone accepted the English language for their official communications. The “Mano River Union” (Fig. 1) was named after the Mano River in West Africa which originates in the Guinea Highlands and forms part of the Liberia-Sierra Leone border [21, 22]. Despite the continued efforts by the MRU governments over the last decades to stabilize the region, it remains unstable as a result of porous borders which facilitate unhindered movement of armed and criminal groups [20, 22].

These countries and areas were selected for this study because of their similarities in social culture, ethnicity and demographic patterns (Fig. 1). Despite the different languages, the four countries have similar geographic and climatic conditions [11, 21, 23, 24]. Because of their contiguity, past epidemics of some diseases or disasters in one country were seen as bonded and overlapped with its neighbour in the region. This can be corroborated by the outbreak of HIV-2 which was first reported in Cote d'Ivoire and Sierra Leone in the 1980s and later disseminated to Guinea, Liberia and beyond [21, 25, 26]. The Ebola epidemic was the recent episode that originated in Guinea in 2014 incubated and transmitted across, within and beyond the union in which the MRU’s weak healthcare system have been stretched and economies ruined to their “death beds” [21, 27]. With such threatening and perilous experience in these communities, HIV/AIDS would not be an exception to be possibly mutated, reassorted and propagated in the region and transformed quietly into an uncontrollable epidemic if left unchecked. Worse still, HIV-positive individuals can harbour a variety of viruses with resultant virulent variants which can wreak havoc in the already strained global public health systems [28]. This chain of events prompted the inception of this study to determine the spatial patterns and temporal heterogeneity of HIV prevalence and hot spots utilizing DHS data and GIS techniques at the national and sub-national levels. Conditions at the border areas are a potential catalyst for the spread of HIV/AIDS putting the region and the globe at risk [18]. Given the untrammelled links between the four countries and the fluidity of the population movement across borders, the battle against HIV/AIDS cannot be won at the country level [20, 22].

Dwyer-Lindgren et al. employed three models (Generalized Additive Model, Boosted Regression Model and Lasso Regression Model) to predict HIV prevalence in countries in Sub-Saharan Africa in 2019 [29]. In 2020, Katia et al. leveraged mathematical models (Shiny90) to Predict knowledge of HIV status and the efficiency of HIV testing, in Sub-Saharan Africa either [30]. No prediction about combined CCKs about HIV/AIDS has been made for sex-specific interventions to bolster HIV testing uptake in the MRU. This prompted us to fill this knowledge gap by making use of data from population-based surveys to assess trends in spatial and temporal distributions of HIV and predict which combinations of CCKs are correlated with an increase in HIV testing and receiving test results. Several DHS conducted in Sub-Saharan Africa have informed policymakers that comprehensive correct knowledge about HIV/AIDS and HIV testing uptake in many countries are still far below the benchmarks set by UNAIDS at 90% by 2020 [4, 31, 32]. Precision public health provides the knowledge to tailor studies using population-specific data to provide the right intervention to the right population at the right time [33]. Comprehensive correct knowledge about HIV/AIDS empowers adults to know about the epidemic in communities [31, 34], and by extension, prevent pregnant women and children from the disease through voluntary HIV testing and treatment. This research aims to perform an ecological study to determine HIV prevalence patterns and the spatial–temporal heterogeneity of HIV prevalence in the MRU.

## Methods

### Study area and data sources

The “Mano River Union” (Fig. 1) was named after the Mano River in West Africa which originates in the Guinea Highlands and forms part of the Liberia-Sierra Leone border. The MRU comprises Cote d’Ivoire, Guinea, Liberia and Sierra Leone [35–38]. See Additional file 1 for more information on the method and data.

In this study, we utilized DHS and AIDS Indicator Survey (AIS) data at the DHS STATcompiler database [39], and their corresponding spatial data at the DHS Spatial Data Repository which are available in shapefiles and geodatabase format [40, 41]. The DHS and AIS are nationally representative cross-sectional surveys in which data are collected for a wide range of health indicators [9].

In this study, we performed an ecological study utilizing DHS and AIS data to describe HIV prevalence, HIV testing uptake and CCK about HIV/AIDS at the population level and location concerning to age, sex, and socioeconomic status of respondents. In this sense, we compared our parameters within and between countries, and over time. Our data are aggregated and designed for groups of adult males and females aged 15–49 years.

The DHS STATcompiler is a tool designed for comparisons across countries and over time. Participating countries’ data are recalculated to match standard definitions. These recalculations are made to meet different time frames, different denominators and country-specific definitions for researchers to be able to compare survey data across countries and over time. However, for researchers who are interested in one single data point, from 1 year and one country, the final DHS report is the best source [40].

The DHS Spatial Data Repository (SDR) and STATcompiler provided the information used in creating the shapefiles with DHS data. To maintain respondents’ confidentiality, the centres of survey clusters are displaced to about 0–2 km for urban clusters and 0–5 km for rural clusters [40]. Then 10% of all survey clusters were further displaced to 10 km (masking). Even though the resulting data is affected by scale and modifiable areal unit problem (MAUP), linking displaced data to very smooth surfaces will likely have little impact on analysis results because covariate values obtained from displaced data will be very similar to those associated with the true, non-displaced location.

In DHS, participants were asked about their comprehensive knowledge about HIV, knowledge of prevention methods of HIV, misconceptions about HIV/AIDS, and their accepting attitude towards people living with HIV etc. Respondents were further asked whether they had been tested for HIV, and when the last test was conducted. The outcome of interest was self-reporting of undergoing an HIV test and receiving test results in the last 12 months. Eligible respondents in the subsample of the selected households were then tested for HIV.

### Data analysis

#### Spatial distribution of HIV prevalence by country and region

We constructed Choropleth maps employing ArcGIS software 10.4 to show temporal variations in the spatial distribution of the regional HIV prevalence among adults from 2005 to 2020. ArcGIS version 10.4 is developed by the Environmental Systems Research Institute (ESRI) in California, United States of America (USA). Utilizing the same GIS software, we also build choropleth maps to monitor the spatial distribution patterns of women with secondary education or higher. We further utilized Spearman Rank correlation (rho) in SPSS version 26 to measure the association between HIV prevalence and women with secondary education or higher. The Statistical Package for Social Sciences (SPSS) was developed by SPSS Incorporated in Chicago, USA and is now owned by the International Business Machines (IBM) in New York, USA.

#### Spatial distribution of HIV hot spots and spatial stratified heterogeneity (SSH) analyses

In this study, we used the index of global Moran’s *I* to identify the spatial autocorrelation of HIV prevalence in MRU countries. We employed the Getis Ord Gi* statistic as the index of local spatial autocorrelation to identify statistically significant spatial clusters of high HIV prevalence (hot spots) and clusters of low HIV prevalence (cold spots) in the MRU countries. To achieve this, the DHS data were divided into three groups (2005–2010, 2011–2015 and 2016–2020) and downloaded as shapefiles format from the DHS Spatial Data Repository. The Getis-Ord Gi statistic provides us with the *Z-score*, *P*-value, and confidence level bin (Gi_Bin). The *Z-scores* and *P* values are measures of statistical significance that tell us whether or not to reject the null hypothesis.

Moreover, we utilized the Geodetector software (www.geodetector.cn) to calculate the index of q-value for the SSH feature for HIV prevalence in the MRU. If the q-value is 0, there is an absence of stratified heterogeneity (SH), and a q-value of 1 represents perfect SH between strata. To perform this analysis, the spatial data were divided into strata factors; country name, region, and survey year. The dominant strata dividing the HIV prevalence in the MRU would be selected from these factors with the highest significant q-value.

### Statistical analysis

We leveraged data on the knowledge about HIV/AIDS, accepting attitudes toward people living with HIV, Knowledge of prevention methods from HIV, knowledge of prevention of mother-to-child transmission of HIV, and misconceptions about HIV etc., which we collectively referred to as comprehensive correct knowledge (CCK) about HIV/AIDS in this study for ease of analysis (Additional file 1: Tables S2 and S3). We utilized the Least Absolute Shrinkage and Selection Operator (LASSO) to predict which combinations of CCK that will be accompanied by an effective increase in voluntary HIV testing and receiving test results. We targeted two testing indicators; adults who ever tested for HIV and received test results (T2) and adults receiving an HIV test and receiving test results in the last 12 months preceding the survey (T6) (Additional file 1: Tables S2 and S3). LASSO is a regularized Gaussian linear regression. R software version 4.1.2, and the Generalized Linear Model via Penalized Maximum Likelihood (*glmnet* 2.0-18) were employed to automatically select the best combination of CCKs (CCK 1–18) that can predict the ratio of men/women to perform T2 and T6 (Additional file 1: Tables S2 and S3). The regularization parameter γ was selected to maximize the area under the curve (AUC) with tenfold cross-validation and the value of alpha (α) was always kept at 1. The largest value of gamma (γ) found within one standard error of γ with the highest AUC (known as lambda.1se) was selected as the final model, as it has fewer parameters than the best model and its accuracy was comparable to the best model. To test the stability and accuracy of the final model, we repeat the above analysis 1000 times. The recurrence rate of a parameter that appears in the final models of these 1000 repetitions was used to assess the stability of this parameter. Only parameters with a recurrence rate > 80% were considered robust parameters in the final model as presented in (Table 5). R software was created by Ross Ihaka and Robert Gentleman in Auckland, New Zealand. It is currently developed by the R Development Core Team.

LASSO is a machine learning method [42] which performs both variable selection and regularization to enhance the interpretability of the statistical results. The glmnet is a package in R software [43] that fits the generalized linear model and other similar models via penalized maximum likelihood. It fits linear, logistic, multinomial, Poisson, and cox, regression models. The package is composed of methods of prediction and plotting, and functions for cross-validation.

The problem below can be solved by glmnet

$$\mathop {\min }\limits_{\beta 0}, \frac{1}{N}\sum_{i-1}^{N}{w}_{i}l\left({y}_{i},{{\beta }_{0}+{\beta }^{T}{x}_{i}}\right)+\lambda \left[(1-\alpha) {\Vert \beta \Vert_2^2}/2+\alpha \Vert {\beta} \Vert {1}\right]$$

In the formula above, *l*(*y*_*i*_, η_*i*_) is the negative log-likelihood contribution of observations i; the elastic net penalty is controlled by α, and bridges the gap between LASSO regression (α = 1), which is the default, and ridge regression (α = 0).

A detailed description of LASSO can be found in Additional file 1 (S1.3).

## Results

### Distribution of CCK and the regional spatial patterns of HIV prevalence

The urban–rural residence has been implicated in the response rates in all the DHS included in this study. Rural residences are accompanied by a higher response rate than urban residences for both adult women and men (Additional file 1: Table S1). While the CCK about HIV, HIV testing uptake, and the knowledge of prevention of mother-to-child transmission of HIV is lower in the rural residence than the urban ones, HIV prevalence is higher in urban residences than their rural counterparts (Tables 1, 2).

**Table 1:** The specific percentages of comprehensive correct knowledge about HIV/AIDS, HIV testing and prevalence by spatial–temporal-population distribution

Countries and DHS year	Comprehensive correct knowledge about AIDS		Knowledge of prevention of mother to child transmission of HIV		Adults ever tested for HIV and received test results		Adults receiving an HIV test and receiving test results in the last 12 months before the survey		HIV prevalence	
	Urban	Rural	Urban	Rural	Urban	Rural	Urban	Rural	Urban	Rural
Women										
Cote d'Ivoire 2005 AIS	18	14.4	45.4	29.7	14.9	7.4	5	2.5	7.4	5.5
Cote d'Ivoire 2012 DHS	20.6	6.9	64	39.6	45.1	24.2	18.5	9.3	5.5	3.6
Guinea 2005 DHS	22.7	11.2	10.5	6.8	5.1	0.7	2.7	0.4	4	0.9
Guinea 2012 DHS	28.8	14.1	37.7	21.5	20.9	4.6	10.1	1.7	3.6	1.3
Guinea 2018 DHS	31.7	13.8	38.7	24.7	28.8	10.8	15.9	4.9	2.4	1.2
Liberia 2007 DHS	27.8	13.2	15.5	9.9	5.3	1.7	2.7	0.9	2.8	1.1
Liberia 2013 DHS	43.1	26.5	57.8	41.3	49.1	38.8	19.9	17.9	2.7	1
Liberia 2019 DHS	37.4	26.9	51.3	44.2	52.8	46.2	23.2	20.2	NA^b^	NA^b^
Sierra Leone 2008 DHS	25.5	7.5	20.3	8.3	18.8	4.2	8.2	1.8	2.7	1.2
Sierra Leone 2013 DHS	38	17.9	62	42.1	43.3	35.6	16.7	11.8	2.5	1.2
Sierra Leone 2019 DHS	35.2	22.2	60.6	45.8	48.7	39.5	24.1	15.3	3	1.5
Men										
Cote d'Ivoire 2005 AIS	32.4	20.3	34.8	25	NA	NA	4.7	1.9	3.2	2.5
Cote d'Ivoire 2012 DHS	33	15.9	46.4	37.9	30.9	15.2	13.4	5.5	3	2.4
Guinea 2005 DHS	29.9	13.8	26.2	18.2	10.7	2.9	5.2	1.4	0.7	1.1
Guinea 2012 DHS	42.6	27.4	26.2	18.2	19.4	6.9	7.9	2.8	1.5	1.1
Guinea 2018 DHS	36	24.1	35.4	23.7	14.9	4.3	9.3	2.1	1.5	1.1
Liberia 2007 DHS	42.5	24.3	19.2	10.1	8.2	2.6	4	1.1	2.1	0.6
Liberia 2013 DHS	41.8	22.9	31.6	20.3	27.4	16.9	15.9	7.6	2.5	0.7
Liberia 2019 DHS	41.6	24.7	31.2	32.6	33.5	29.9	22.6	18.9	NA	NA
Sierra Leone 2008 DHS	38.8	16.3	27	15.4	13.2	3.2	6.5	1.6	2.2	0.6
Sierra Leone 2013 DHS	36.5	28.1	36.2	28.3	22	9.6	9.3	4.3	2	0.8
Sierra Leone 2019 DHS	39.4	27.7	38	34.1	27.1	14	16.3	8	1.5	0.7

*NA* data not available, *DHS* Demographic and Health Surveys, *HIV* Human Immunodeficiency Virus, *AIDS* Acquired Immune Deficiency Syndrome, *AIS* AIDS Indicator Survey, *USAID* United States Agency for International Development

^a^ICF, 2015. The DHS Program STATcompiler. Funded by USAID. http://www.statcompiler.com. February 15 2022 [39]

**Table 2:** Percentage HIV prevalence and prevalence of women with secondary education or higher among adults aged 15–49 years by regions in the MRU

Country name and regions	Women with secondary education and higher	HIV prevalence among women with secondary education and higher	Women with secondary education and higher	HIV prevalence among women with secondary education and higher	Women with secondary education and higher	HIV prevalence among women with secondary education and higher
Cote d'Ivoire	2005 AIS	2005 AIS	2012 DHS	2012 DHS		
Abidjan	32.7	8.6	37.3	5.9	NA	NA
Central	22.3	5.1	21.6	3.7	NA	NA
East Central	12.5	8.6	23.7	5.1	NA	NA
North Central	12.1	4.2	18.6	6.3	NA	NA
West Central	19.3	5.5	16.8	3	NA	NA
North	9	4.2	12.8	3.3	NA	NA
Northeast	6.6	4.4	14.5	2.4	NA	NA
Northwest	5.2	2.7	5.1	2.4	NA	NA
West	8.2	4.6	12.1	4.9	NA	NA
South	19.7	8	21.4	4.3	NA	NA
Southwest	13.5	5	13.3	5.2	NA	NA
Guinea	2005 DHS	2005 DHS	2012 DHS	2012 DHS	2018 DHS	2012 DHS
Conakry	33.6	3.6	47.7	3.5	49.3	2.1
Bonke’	7.8	1.4	19.1	1.9	17	2.3
Faranah	8.4	2	7	1.5	10.4	1.7
Kankan	3.8	1.2	6.9	1.5	11.9	1
Kindia	7	1	13.7	1.4	17.4	1.4
Labe’	6.7	1.7	11	1.4	10.8	2
Mamou	4.8	1.2	8.2	1.5	10.3	1.3
N’Ze’re’koreh	9.1	2.2	13.6	2.5	12.9	1.2
Liberia	2007 DHS	2007 DHS	2013 DHS	2013 DHS	2019 DHS	2019 DHS
Monrovia	50.2	2.9	60.2	NA	70.7	NA
North Western	11.1	2	16.6	1.2	25.3	NA
South Central	14.8	2.2	49.3	2.6	60.6	NA
South Eastern A	9	1.4	20.3	1.1	25.6	NA
South Eastern B	12.7	2.4	24.6	2.7	30.2	NA
North Central	11.6	0.5	21.3	1.2	32.2	NA
Sierra Leone	2008 DHS	2008 DHS	2013 DHS	2013 DHS	2019 DHS	2019 DHS
Eastern	13.6	1.6	24.6	1.7	35.8	1.8
Northern	12.1	1.4	23.6	1.4	33.3	1.9
Southern	13.6	1.1	51.4	2.2	33.5	2.1
Western	53.1	3.1	NA	NA	64.7	3.1
North Western	NA	NA	NA	NA	30.7	1.8

Table represents the regional percentages of women with secondary education or higher and their corresponding regional HIV prevalence among adults women age 15–49 years. It shows the importance of focusing HIV programs in the MRU on women with higher social status in communities

*MRU* Mano River Union, *NA* data not available, *DHS* Demographic and Health Surveys, *HIV* Human Immunodeficiency Virus, *AIDS* Acquired Immune Deficiency Syndrome, *AIS* AIDS Indicator Survey, *USAID* United States Agency for International Development

^a^ICF 2015. The DHS Program STATcompiler. Funded by USAID. http://www.statcompiler.com. August 4 2022

**Fig. 2:**
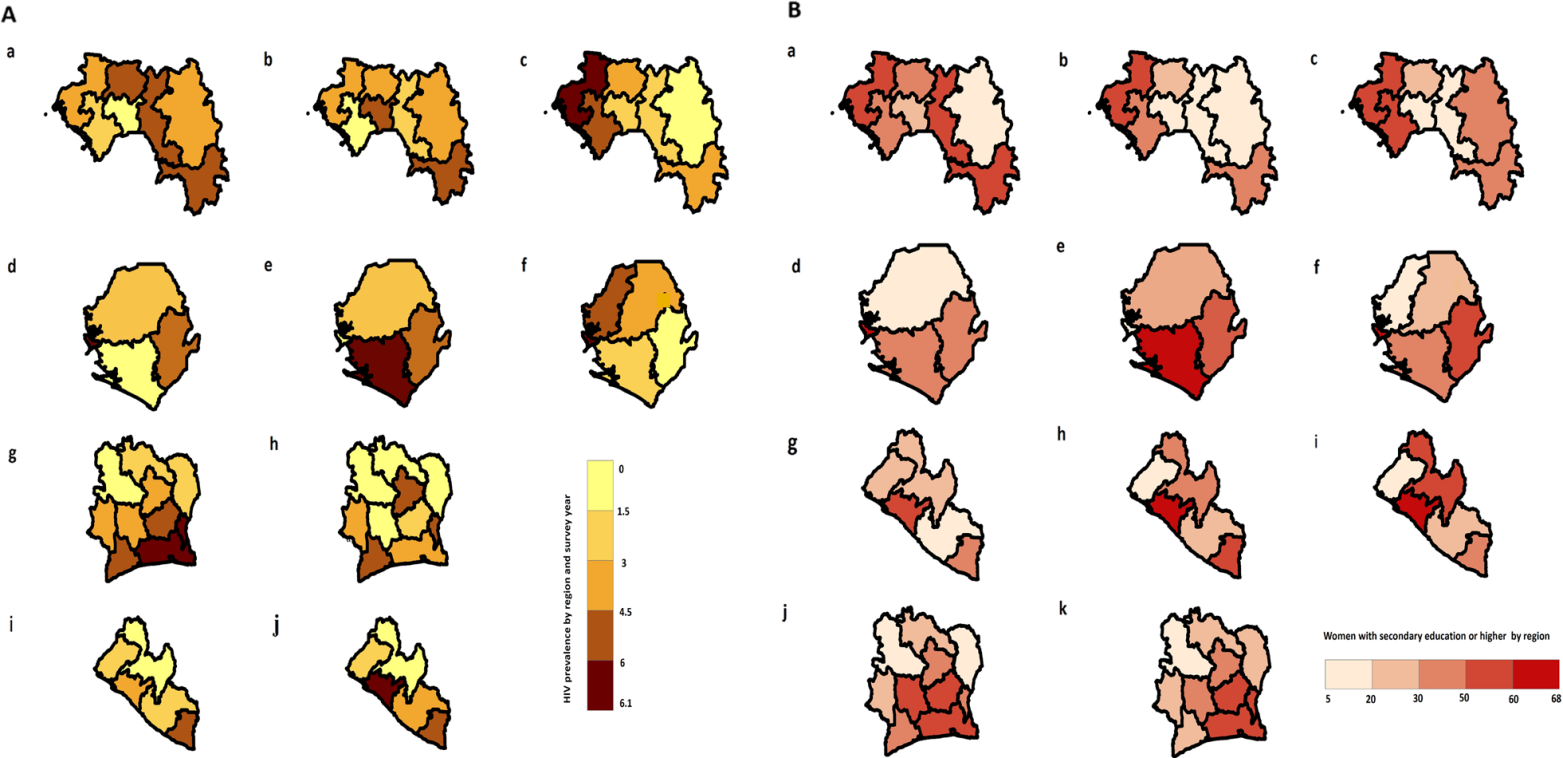
HIV prevalence in the Mano River Union countries. **A** Represents choropleth maps for the spatial variation of HIV prevalence in the general population among adults aged 15–49 years by country and region concerning DHS. **a**–**c** Guinea 2005, 2012, and 2018 DHS respectively; **d**–**f** Sierra Leone 2008, 2013, and 2019 DHS respectively; **g** Cote d’Ivoire 2005 AIS, **h** Cote d’Ivoire 2012 DHS; **i**, **j** Liberia 2007 and 2013 DHS respectively. The Demographic and Health Survey Program. ICF International. Available from spatialdata.dhsprogram.com (26 November 2020) [41]. **B** represents choropleth maps for the spatial variation of adult women aged 15–49 years with secondary education or higher by country and region concerning DHS. **a**–**c** Guinea 2005, 2012, and 2018 DHS respectively; **d**–**f** Sierra Leone 2008, 2013, and 2019 DHS respectively; **g**–**i** Liberia 2007, 2013 and 2019 DHS respectively; **j** Cote d’Ivoire 2005 AIS, **k** Cote d’Ivoire 2012 DHS; The Demographic and Health Survey Program. ICF International. Available from the spatialdata.dhsprogram.com (26 November 2020) [41]. *HIV* Human Immunodeficiency Virus, *DHS* Demographic and Health Surveys, *AIS* AIDS Indicator Survey, *ICF* International Classification of Functionality, Disability and Health

Among the four countries, Cote d’Ivoire, Guinea, Liberia and Sierra Leone have eleven, eight, six and four DHS regions respectively, as their administrative or subnational level 1. In Cote d’Ivoire 2005 AIS and 2012 DHS, the capital, Abidjan, has the highest HIV prevalence. In the 2005 AIS, the HIV prevalence is concentrated in Abidjan and its neighbouring regions (South, East Central, Central and Southwest), while Northwest has the lowest. In 2012 DHS, the HIV prevalence is highest in North Central, East Central and Southwest regions. Conversely, the lowest HIV prevalence occurs in the Northwest, North, West Central, and Northeast regions (Fig. 2A; g, h).

Guinea has 3 DHS from 2005 to 2020 in this analysis. The capital city, Conakry, has the highest HIV prevalence for two DHS (from 2005 to 2015). In 2005 DHS, Labe, Faranah and N'zerekore have the highest HIV prevalence, while Momou and Kindia have the lowest. In 2012 DHS, HIV prevalence was more prominent in Mamou and N'zerekore regions, and less prominence of HIV prevalence was seen in Kindia and Faranah regions. 2018 DHS is accompanied by unique features wherein HIV prevalence was highest in Boke and Kindia, and the lowest is seen in Kankan, Faranah and Mamou regions. Conakry city is overtaken by Boke region in HIV prevalence (Fig. 2A; a–c).

In Liberia, the regional HIV prevalence follows a definite pattern. In 2007 DHS, Monrovia, the capital, was considered as a region on its own with the highest HIV prevalence followed by the South Eastern B region. In the 2013 DHS, Monrovia was incorporated in the South Central region which led the region to have the highest HIV prevalence and again followed by South East B. In both surveys, North Central has the lowest HIV prevalence in the country (Fig. 2A; i, j). The Liberia 2019 HIV prevalence data were not made public, notably; it was exempted from our spatial analysis.

The Sierra Leone 2008 and 2013 DHS have regional variations of HIV prevalence over time. In 2008 DHS, the Western Area where the capital city, Freetown, is located is seen with the highest HIV prevalence followed by the Eastern Region. In contrast, the Southern Region has the lowest HIV prevalence in that survey year. In 2013 DHS, the Western region has no available data on HIV prevalence; consequently, the Southern region takes the lead this time, while the Northern region took the lowest HIV prevalence. In 2019 DHS, Sierra Leone has five DHS regions, thus, HIV prevalence is concentrated in the Northwest and Western region. Conversely, the Eastern region has the lowest HIV prevalence (Fig. 2A; d–f).

### The spatial distribution of HIV prevalence and women with secondary education or higher

Investigating the spatial distribution of “women with secondary education or higher” follow similar patterns and dynamics to that of HIV prevalence in the general population among adults aged 15–49 years. Our correlation analysis between HIV prevalence and women with secondary education or higher revealed a weak positive linear correlation of 0.32 and a *P-value* of 0.01, thus indicating there is a weak significant relationship between HIV prevalence and women with secondary education or higher in the MRU (Fig. 2B; a–k). People with secondary education or higher typically have higher comprehensive correct knowledge about HIV/AIDS and higher HIV testing uptake than their lower and non-educated peers (Tables 1, 2). Locating such groups in communities and studying their spatial patterns and dynamics can reveal changes in HIV interventions and foster improvements. There is a policy implication for this group of women. In a situation where women with secondary education or higher have unacceptably high percentages of HIV prevalence among women aged 15–49 years, targeting higher social classes among women in HIV interventions is as important as continuing education to increase HIV knowledge. This is a worrying sign that the gravity of HIV disease and its implication for our behaviour has not been fully grasped. HIV prevalence in Africa is higher among women than among men and the main focus of many HIV programs is on females [44, 45].

### HIV hot spots and stratified heterogeneity (SH) by gender and country level

**Fig. 3:**
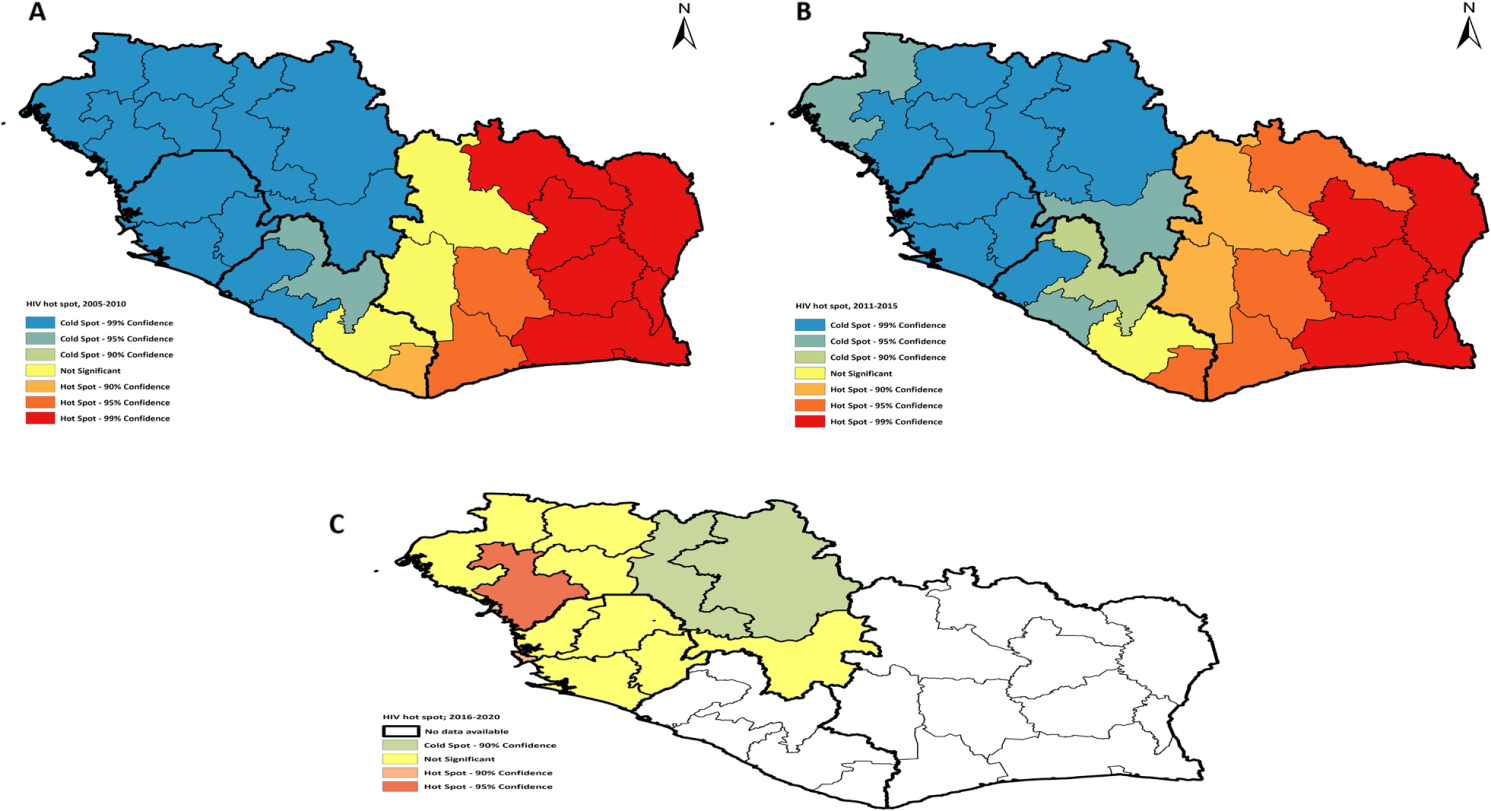
HIV significant spatial clusters of high HIV prevalence (hot spots) and low HIV prevalence (cold spots) in the MRU countries for 15 years (2005–2020). **A** Represents the HIV hot spots in the general population for the first group of our geostatistical analysis from 2005 to 2010 concerning DHS. **B** Represents the HIV hot spots in the general population for the second group in our geostatistical analysis from 2011 to 2015 concerning DHS. **C** Represents the HIV hot spots in the general population for the third group in our geostatistical analysis from 2016 to 2020 concerning DHS. Spatial Data Repository; The Demographic and Health Surveys Program. ICF International. Available from spatialdata.dhsprogram.com (26 November 2020) [41]. *HIV* Human Immunodeficiency Virus, *MRU* Mano River Union, *DHS* Demographic and Health Surveys, *ICF* International Classification of Functionality, Disability and Health

In the first and second 5 years of this study (2005–2010, 2011–2015), Abidjan, South, Southwest, Central, East Central, North Central, West and West Central regions in Cote d’Ivoire are the hot spots for HIV prevalence, with 99, 95 and 90% confidence, Gi_Bin Score of 3, *Z*-Score of 3.77–3.93 and *P* < 0.01 (Fig. 3A, B), while Guinea, Liberia and Sierra Leone are the cold spots for HIV with 90, 95, and 99% confidence. In the third 5 years of this study (2016–2020), only Guinea and Sierra Leone have data on HIV prevalence. Conakry and Kindia regions in Guinea and the Western region in Sierra Leone are hot spots for HIV with 95, and 90% confidence, Gi_Bin Score of 2, *Z*-Score of 3.12, and *P* < 0.01 (Table 3). All other regions are cold spots and spots of no significant (Fig. 3C).

**Table 3:** Summary of geospatial peaks scores for HIV hot spot in the MRU

Years	Peaks	Distance value	Moran’s *I*	Expected index	Variance	*Z*-score	*P*-value	Gi-Bin scores
2005–2010	First peak	446,971.00	0.497	− 0.037	0.003	9.87	< 0.01	3
	Maximum peak	574,677.00	0.356	− 0.037	0.002	10.09	< 0.01	3
2011–2015	First peak	446,971.00	0.416	− 0.037	0.003	7.89	< 0.01	3
	Maximum peak	574,677.00,	0.301	− 0.037	0.002	7.89	< 0.01	3
2016–2020	One peak	344,924.18	0.095	− 0.083	0.003	3.12	< 0.01	2

With the use of ArcGIS software 10.4, this statistically significant results is the output for Incremental Spatial Autocorrelation tool that runs Global Moran’s *I* and Getis-Ord GI* tool in our hot spot analyses from 2005 to 2020. The *Z*-scores, *P* values and the confidence level bin (Gi_Bin) scores identify statistically significant hot and cold spot. Gi_Bin of + 3 and + 2 reflect statistical significance with 99% and 95% confidence level respectively. High Z-scores and small P values indicate clustering of high values. Values of the local Moran’s I index indicate statistically significant clustering with no outliers. The statistically significant peak distance value Z-scores indicates distances where spatial processes promoting clustering are pronounced

*MRU* Mano River Union, *HIV* Human Immunodeficiency Virus

^a^Global Moran’s *I* summary by distance in (m) and Gi_Bin scores

In our Geodetector statistical analysis, the strata in the MRU were measured for their interactions and risk factors in HIV prevalence concerning gender. The q-values and p-values indicate that there are significant differences in risk factors at the country level than at the regional level. Women in Cote d’Ivoire had the highest risk for HIV prevalence. Additional significant risk is seen in the Cote d’Ivoire 2005 AIS for women (Table 4). Among adult men, there is no significant difference in these four factors.

**Table 4:** The spatial stratified heterogeneity of HIV prevalence among adult women and men aged 15–49 years

Gender group	Factors or interaction factors	*q*-value	*P*-value	Highest risk points	Ecological detector
Women	Country	0.61	0.000	Cote d'Ivoire	There is significant difference between these four factors
	Year	0.29	0.204	2005	
	Region	0.57	0.002	Abidjan	
	Country and year	0.66	0.002	Abidjan	
Men	Country	0.40	0.000	Cote d'Ivoire	There is no significant difference between these four factors
	Year	0.20	0.914	2005	
	Region	0.37	0.037	Abidjan	
	Country and year	0.42	–	–	

SSH were analyzed by the “Geodetector” software with *q*-value from 0 to 1. If *q* = 0, the spatial heterogeneity is not significant between strata, or vice versa. In this study, country was a dominant factor contributing to different HIV prevalence among women and men aged 15–49 years. Moreover, the interaction effect of country and year shows higher heterogeneity. However, there is no significant difference between strata factors for HIV prevalence among men 15–49 years [19]

*HIV* Human Immunodeficiency Virus

### HIV temporal changes

**Fig. 4:**
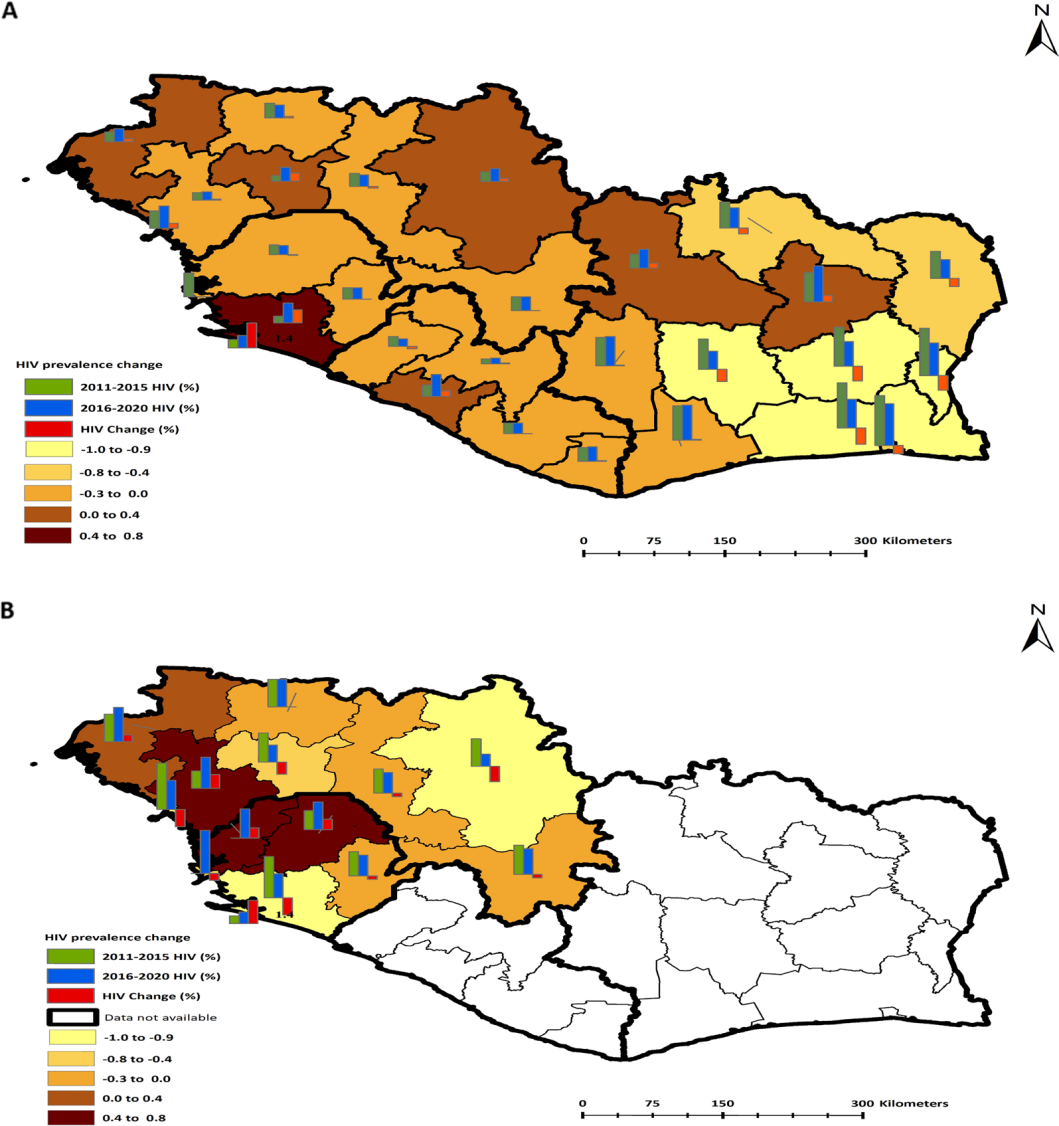
Represents temporal changes in HIV prevalence in the MRU countries from 2005 to 2020. **A** The bar charts in the map represent changes in HIV prevalence by region. The green bar represents 2005–2010 HIV prevalence in the first DHS, the blue bar represents 2011–2015 HIV prevalence in the second DHS, and the red bar represents the changes (increase or decrease) in HIV prevalence. **B** The green bar represents 2011–2015 the second DHS, the blue bar represent 2016–2020 the third DHS, and the red bar represents the changes (increase or decrease) in HIV prevalence. Spatial Data Repository, The Demographic and Health Surveys Program. ICF International. Available from *spatialdata.dhsprogram.com* (26 November 2020) [41]. *HIV* Human Immunodeficiency Virus, *MRU* Mano River Union, *DHS* Demographic and Health Surveys, *ICF* International Classification of Functionality, Disability and Health

Examining spatial–temporal changes in HIV prevalence for 15 years revealed unique features of the disease in the MRU countries. While other regions see a percentage decrease in HIV prevalence, some regions are accompanied by a surge in HIV prevalence. In Cote d'Ivoire for example, in the North Central, North West, South West and West regions, the proportions of adults in the general population with HIV prevalence increased by 0.8, 0.6, 0.1, and 0.1% respectively (Fig. 4a). From 2005 to 2015, Cote D’Ivoire saw a 1.6% total increase in HIV prevalence in four regions and a 9.8% total decrease in HIV prevalence in seven regions.

Guinea has eight DHS regions from 2005 to 2015. During this period, there is a 2.7% increase in HIV prevalence in five regions and a 0.4% decrease in HIV prevalence in two regions. However, the percentage of HIV prevalence in one region (Nzerekore) remains at 1.7% from 2005 to 2015 (Fig. 4A). In 2018 DHS, Boke has the highest HIV prevalence, followed by Kindia and Conakry by 2%, 1.8% and 1.7% respectively. The lowest HIV prevalence is seen in the Kankan region at 0.7%. Overall, the Guinea 2018 DHS saw a decrease in HIV prevalence in five regions, with an opposing increase in two regions (Boke and Kindia), and Labe which remains unchanged at 1.6% (Fig. 4B).

Liberia has six DHS regions in the 2007 DHS and five DHS regions in the 2013 survey. In the 2007 DHS, the capital city, Monrovia, was treated as a region on its own, while in the 2013 DHS, Monrovia was incorporated into the South Central region. After the adjusted analysis for the differences in DHS regions, Liberia has a 0.7% increase in HIV prevalence in three regions and a 0.3% decrease in HIV prevalence in one region. The HIV prevalence in South East A region remains at 1.3% from 2005 to 2015 (Fig. 4A).

Sierra Leone has four DHS regions for the 2008 and 2013 DHS. However, the re-demarcation of the country at the admin 1 and admin 2 levels in 2011 altered the regional boundaries in the Western region of the country. As a result, Spatial Data Repository and STATcompiler do not have data for the Western Region in the 2013 DHS for HIV prevalence. Changes in HIV prevalence follow a similar pattern with sister countries in the region. There is a 1.6% increase in HIV prevalence in the Southern Region and a 0.1% decrease in HIV prevalence in the Northern Region. The Eastern Region remains at 1.4% from 2005 to 2015 (Fig. 4A). In Sierra Leone 2019 DHS, the country has five DHS regions. The Northern region is divided into Northwest and Northern regions. After the adjustment by averaging the two new regions, the Northern region has a surge of 0.6% in HIV prevalence. Overall, there are decreases in HIV prevalence in Eastern, Southern and Western regions with 0.2%, 1.0% and 0.4% respectively. At the national level, Cote D'Ivoire has done better than all the other countries in the MRU in reversing the HIV epidemic by having the highest number of DHS regions with a decrease in HIV prevalence (7 out of 11) at 9.8% from 2005 to 2015. Only four regions have an increase in HIV prevalence with 1.6%. Despite this, the country remains the hot spot for HIV in the MRU. Guinea is the least performing country in reversing the HIV epidemic from 2005 to 2015 in which there is an increase in HIV prevalence in five regions (5 out of 8) with 2.7%. Two regions have a decrease in HIV prevalence with only 0.4%. One region remains unchanged from 2005 to 2015. Liberia is a lower performer in reversing the HIV epidemic with a surge in HIV prevalence in three regions with 0.7% and a decrease in only one region with 0.3% from 2005 to 2015. Sierra Leone has an increase in HIV prevalence in one region (1 out of 4) by 1.6% and a decrease in one region by 0.1% from 2005 to 2015. In the last 5 years of this study (2016–2020), only Guinea and Sierra Leone have data on HIV (Fig. 4B). Ultimately, Guinea made inroads by a decrease in HIV prevalence in 6 regions and an increase in only 2 regions. Sierra Leone has an increase in HIV prevalence in one region and a decrease in 3 regions.

### The combinations of predicted CCK sets

Improving voluntary HIV testing in the MRU can further increase case finding and case identification. In this regard, our model predicted different combinations of CCKs when targeting different populations in this community. From the LASSO result, we could find that the CCK12 (prevention knowledge of mother-to-child transmission (MTCT)-(HIV can be prevented by mother taking special drugs during pregnancy) was the only CCK that positively contributed to the ratio of both men and women “ever tested for HIV and received test results” (T2) and “receiving an HIV test and receiving test results during the last 12 months preceding the survey” (T6) (Table 5), indicating its significant importance in these four countries. When we paid attention to which set of CCK related to enhancing the ratio of men receiving HIV testing, we found that CCK7-9, CCK12, CCK14, CCK17, and CCK18 all contributed to the ratio of men ever tested for HIV and received test results (T2). However, only CCK10 (no incorrect beliefs about AIDS—composite of 3 components) and CCK12 positively contributed to the ratio of men receiving an HIV test and receiving test results during the last 12 months before the survey (T6) (Table 5). This result suggested the ratio of men receiving an HIV test and receiving test results during the last 12 months preceding the survey could be easily increased by popularizing CCK10 and CCK12 in these four countries. For women, the CCKs that contributed to the ratio of receiving an HIV test were different from those for men. CCK7, CCK12, CCk13, and CCK15 all positively contributed to the ratio of women ever tested for HIV and received test results (T2) (Table 5). While, CCK7, CCK12, and CCK13 all positively contributed to the ratio of women receiving an HIV test and receiving test results during the last 12 months before the survey (T6) (Table 5). All these results suggested that different emphases are needed when popularizing the comprehensive correct knowledge about HIV/AIDS for men and women to increase the ratio of receiving HIV tests and getting the result.

**Table 5:** Predicted CCK among adults aged 15–49 years in the MRU countries

Parameter	Detailed parameter description	Recurrence rate (%)	Mean	95% *CI*
	Adult women aged 15–49 ever tested for HIV and received test results			
Intercept		100%	− 7.15	(− 10.35, − 4.44)
CCK12	Prevention knowledge of MTCT—can be prevented by mother taking special drugs during pregnancy	100%	0.21	(0.19, 0.23)
CCK13	Knowledge of prevention of mother to child transmission of HIV	100%	0.34	(0.31, 0.39)
CCK15	Accepting attitudes—would buy fresh vegetables from a shopkeeper with AIDS	91.30%	0.01	(0.002, 0.03)
CCK7	No incorrect beliefs about AIDS—AIDS cannot be transmitted by mosquito bites	100%	0.16	(0.12, 0.19)
	Adult men aged 15–49 who ever tested for HIV and received test results			
Intercept		100%	− 6.32	(− 7.39, − 1.90)
CCK12	Prevention knowledge of MTCT—can be prevented by mother taking special drugs during pregnancy	100%	0.26	(0.26, 0.27)
CCK14	Accepting attitudes—willing to care for family member sick with AIDS	92.10%	0.02	(0.01, 0.03)
CCK17	Accepting attitudes—Not secretive about family member's HIV status	96.50%	− 0.09	(− 0.11, − 0.01)
CCK18	Accepting attitudes towards those living with HIV- composites of 4 components	92.10%	0.18	(0.08, 0.24)
CCK7	No incorrect beliefs about AIDS—AIDS cannot be transmitted by mosquito bites	92.70%	0.01	(0.01, 0.02)
CCK8	No incorrect beliefs about AIDS—AIDS cannot be transmitted by supernatural means	88.70%	0.06	(0.01, 0.09)
CCK9	No incorrect beliefs about AIDS—cannot become infected by sharing food with someone who has AIDS	100%	0.12	(0.09, 0.14)
	Adult women aged 15–49 receiving an HIV test and receiving test results in last 12 months before the survey			
Intercept		100%	− 1.86	(− 3.38, − 0.29)
CCK12	Prevention knowledge of MTCT—can be prevented by mother taking special drugs during pregnancy	100%	0.16	(0.15, 0.16)
CCK13	Knowledge of prevention of mother to child transmission of HIV	99.40%	0.02	(0.0001, 0.04)
CCK5	Comprehensive correct knowledge about AIDS	100%	0.04	(0.04, 0.05)
CCK7	No incorrect beliefs about AIDS—AIDS cannot be transmitted by mosquito bites	99.90%	0.05	(0.03, 0.06)
	Adult men aged 15–49 receiving an HIV test and receiving test results in last 12 months before the survey			
Intercept		100%	− 1.58	(− 3.18, − 0.13)
CCK10	No incorrect beliefs about AIDS—composite of 3 components	100%	0.07	(0.05, 0.08)
CCK12	Prevention knowledge of MTCT—can be prevented by mother taking special drugs during pregnancy	100%	0.13	(0.11, 0.17)

*CCK* Comprehensive Correct Knowledge, *MRU* Mano River Union, *AIDS* Acquired Immune Deficiency Syndrome, *HIV* Human Immunodeficiency Virus, *LASSO* Least Absolute Shrinkage and Selection Operator

^a^LASSO Regression Model: in terms of specific model group, some of the optimal future campaign strategies among a lot of CCKs about HIV/AIDS were predicted by LASSO regression model. This table represents the combinations of predicted CCKs about HIV/AIDS for adults aged 15–49 years with sex specific needs in MRU countries

## Discussion

This study provides a precise quantification of spatial–temporal heterogeneity of HIV prevalence in the MRU nations from 2005 to 2020 and predicts the combinations of CCK that will improve voluntary HIV testing using DHS data. To the best of our knowledge, this is the first study of HIV prevalence in the MRU with geospatial techniques to describe clusters and hot spots of HIV prevalence, and leverage machine learning to predict the combinations of CCKs that will be accompanied by an increase in future HIV testing uptake using DHS data. This is important because the results of this study can be used by policymakers to fine-tune HIV interventions and popularize our predicted CCKs through social and mass media to bolster voluntary HIV testing in the MRU and interrupt the transmission chain to save lives.

Map literacy and map use have immensely increased in the areas of research, project planning, advocacy, monitoring and evaluation of programs [29, 46]. HIV prevalence is higher in urban residents than in rural residents among adults aged 15–49 years in all the surveys included in our study (Tables 1, 2). The stealth behaviour of HIV should be continuously investigated in communities as HIV has the potential to spread quietly unnoticed. For example, the first case of HIV-1 infection in the USSR was detected in the late 1980s, but the expansion of HIV-1 started only in 1986 when a subtype A strain was introduced in people who inject drugs. In 2016, over 100,000 new cases of HIV were reported due to a lack of frequent surveillance because the epidemic was growing rapidly unknown to the authorities [14, 26]. Another astonishing instance is the VB HIV-1 variant which was highly aggressive and contagious that was silently spreading in the Netherlands for decades. Researchers discovered that people infected with the VB variant have higher viral loads, making them more likely to transmit the virus to others [28].

Geodetector is a statistical tool that utilizes q-statistics to measure stratified heterogeneity, dominant driving force detection and an interaction relationship investigation. It involves five functions; risk detector, interaction detector, ecological detector, factor detector and an auxiliary Geodetector [19]. In this study, the country level is the dominant factor dividing HIV prevalence for adult women and men, and Cote d'Ivoire is the highest risk point among MRU countries (Table 4). Additionally, the survey year shows promoting interaction impacting HIV prevalence for adult women and men aged 15–49 years. However, the ecological detector indicates no significant association between the strata factors and HIV prevalence for adult men and the q-value of country strata for adult men aged 15–49 years is lower than for adult women. This possibly resulted from the fact that adult men in MRU were better informed to reach out for CCK and implement more preventive methods against HIV than women (Additional files 2, 3).

In the MRU countries, HIV prevalence is higher among women than among men, and the illiteracy rate is higher among women than among men either (Tables 1, 2) [34]. Narrowing down CCKs about HIV/AIDS to the most important parameters that are best fit for certain nations in surveys and programs can mitigate the fear and stigma among adults for receiving an HIV test and getting test results. The HIV testing uptake is very low in the MRU countries and is decreasing because of COVID-19 [14, 47]. Research on HIV prevalence conducted on Ebola suspected patients during the 2014 Ebola epidemic at the Sierra Leone-China Friendship Biosafety Level 3 Laboratory revealed higher percentages of HIV-positive persons, thus indicating HIV prevalence in these nations might be underestimated due to lower testing uptake [48]. HIV testing is the first 95 of the United Nations General Assembly Political Declaration on HIV/AIDS and it’s a prerequisite for people to determine HIV prevalence and status. This study is informing policymakers and program managers that to increase the HIV testing uptake for adults aged 15–49 years, “ever tested for HIV and received test results” (T2) among women in the MRU, CCKs 7, 12, 13 and 15 are enough to tell them on radio, TVs, meetings etc. with 100% recurrence rates (Table 5). Conversely, our model predicted that CCKs 7, 8, 9, 10, 12, 14, 17 and 18 can bolster the proportion of men who ever tested for HIV and received test results (T2) with up to 90% recurrence rates (Table 5). To improve the ratio of adult women “receiving an HIV test and receiving test results in the last 12 months preceding the survey” (T6), CCKs 7, 12, and 13 are quite sufficient to popularize in antenatal clinics (ANC) and beyond with 100% recurrence rate. On the other hand, CCK 10 and 12 are adequate to inform adult men aged 15–49 years to increase the ratio of “receiving an HIV test and receiving test results in the last 12 months before the survey” (T6) with a 100% recurrence rate (Table 5). Finally, CCK12 (prevention knowledge of mother-to-child transmission (MTCT)-HIV can be prevented by the mother taking special drugs during pregnancy) is the only CCK that positively contributed to the ratio of both men and women and for both T2 and T6 (Table 5). Policymakers and program managers should pay great attention to popularizing this CCK in the MRU because adults in this sub-region are very conscious about the health of pregnancies and that of their newborns.

Previous studies have revealed that secondary education or higher are an important marker for the status of health in countries and communities. There are assumptions that People with secondary education or higher typically have lower odds of contracting HIV/AIDS, especially for women, with resultant better health outcomes and economic development than those with no education or lower [44]. Socioeconomic status such as education, employment and wealth has been reported to be associated with a decrease in HIV prevalence in communities.

HIV has long been known to be the disease of the poor. Illiteracy and low levels of education among women are the vulnerabilities that cause higher HIV prevalence among women than among men in Africa. In a situation like this, early and continuous testing of all social classes with a stamp-out strategy could significantly disrupt the transmission chain in the MRU. The most effective way to reverse the HIV epidemic is to offer HIV testing services at locations and among populations with the highest HIV burden to initiate treatments.

Both the data used in this analysis and the methods are subject to limitations. First, HIV testing uptakes in surveys rely on self-reporting data which is subject to recall bias. Second, DHS HIV prevalence surveys target adult men aged 15 to 59 year and women aged 15–49 years. However, our analysis was limited to aged 15–49 years for both sexes for proper comparison and ease of effective analysis. These might have excluded some HIV-positive cases out of this age range and consequently bias our results. Third, the masking of cluster locations may affect our spatial analyses. Fourth, self-reporting on CCK about HIV/AIDS and HIV testing depend on human cognition and memory, the elaborate nature of CCKs on DHS in post-conflict and fragile nations like the MRU might provide misleading information during the surveys. Notably, our LASSO model might over predict CCK combinations in the MRU.

## Conclusions

Cote d’Ivoire has the highest number of regions with a decrease in HIV prevalence but yet it exists as a hot spot for HIV in the region from 2005 to 2015. At the national level, Guinea, Liberia and Sierra Leone have a high regional increase in HIV prevalence despite relative cold spots. The HIV testing uptake and CCK are far below the benchmarks set by UNAIDS for ending the epidemic, irrespective of residence, sex, age and socioeconomic status. The elaborate CCKs about HIV/AIDS, compounded with the lower literacy rate and instability in the sub-region might have translated into lower knowledge about HIV and HIV testing uptake among adults aged 15–49 years. Further research is needed among individuals out of our age limit and determines why HIV is higher among adults with higher socioeconomic status than their lower peers. Coupled with the above, more work on HIV education is needed in this sub-region to scale up HIV testing uptake for early ART interventions and disrupt the transmission chain.

## Supplementary Information

**Additional file 1.** Spatial and statistical analyses for predicted CCKs.

**Additional file 2.** Stratified Heterogeneity (SH) for HIV risk among women determined by Geodetector.

**Additional file 3.** Stratified Heterogeneity (SH) for HIV risk among men determined by Geodetector.

## Data Availability

The datasets analyzed in our study are publicly available from the DHS website www.DHSprogram.com and public access to the database is restricted. Administrative permission to access the data was obtained.

## Abbreviations

AIDS: Acquired Immune Deficiency Syndrome
AIS: AIDS Indicator Survey
CCK: Comprehensive correct knowledge
DHS: Demographic and Health Surveys
GIS: Geographic Information System
ESRI: Environmental Systems Research Institute
GPS: Geographic positioning system
HIV: Human Immunodeficiency Virus
IBM: International Business Machines
ICF: International Classification of Functionality, Disability and Health
LASSO: Least Absolute Shrinkage and Selection Operator
MRU: Mano River Union
MTCT: Mother-to-child transmission
USAID: United States Agency for International Development
SPSS: Statistical Package for Social Sciences
WHO: World Health Organization
SDR: Spatial Data Repository
INS: Institute of National Statistics
SSL: Statistics Sierra Leone
MEASURE: Monitoring and evaluation to assess and use results
SSH: Spatial stratified heterogeneity
