# Supplementary material for: Spatial–temporal heterogeneity and determinants of HIV prevalence in the Mano River Union countries
Source: Infect Dis Poverty. 2022 Nov 29;11:116. doi: 10.1186/s40249-022-01036-1 (PMC9706865; doi:10.1186/s40249-022-01036-1)
Supplement: Supplementary file 1 — Additional file 1. Spatial and statistical analyses for predicted CCKs. [file 40249_2022_1036_MOESM1_ESM.docx]

**Additional file**

**Spatial-stratified heterogeneity and determinants of HIV prevalence predicted by machine learning in the Mano River Union (MRU) countries**

**Table of Contents**

**Figures:**

**S1 Incremental Spatial Autocorrelation Line Graphs………………………………….…….…….2**

**S1.1 Method and Data …………………………………………………………….………..…..…….3**

**S1.2 Spatial distribution of HIV hot spots and spatial stratified heterogeneity (SSH) analyses………………………………………………………………………4**

**S1.2 HIV Temporal Changes…………………………………………………………………….…4**

**S1.3 Least Absolute Shrinkage and Selection Operator………………………….……..……….…5**

**Tables:**

**Table S1 Sample sizes and response rate for different DHS in the MRU for 15 years……….8**

**Table S2 LASSO Regression Model for Women………………………………….……..……9-11**

**Table S3 LASSO Regression Model for Men………………………………………………..12-14**

**Tables S4a-S7g Geodetector analyses for SSH……………Excel additional file for women**

**Tables S8a-S11g Geodetector analyses for SSH…………..Excel additional file for men**

**^a^Figure S1. Incremental spatial autocorrelation line graphs for 2005 - 2020 hot spot analyses**

**^a^A= 2005-2010, B=2011-2016, C=2016-2020**

**^b^**With the use of ArcGIS software 10.4, this statistically significant results are the output line graphs for Incremental Spatial Autocorrelation tool that runs Global Moran’s I and Getis-Ord GI* tool in our hot spot analyses from 2005 to 2020. The Z-scores, identify statistically significant hot and cold spot.. High Z-scores and small P values indicate clustering of high values. The statistically significant peaks and distance value Z- scores indicates distances where spatial processes promoting clustering are pronounced.

**S1.1 Method and data**

The (Demographic and Health Survey) DHS and AIDS Indicator Survey (AIS) are household based surveys. The DHS program main objective is to enhance the collection, analysis, and dissemination of population health, and nutrition data and to expedite use of these data for research, planning, policy-making and program management. The DHS created the AIDS Indicator Survey (AIS) to respond to the need for universal monitoring of the HIV/AIDS epidemic. The AIS focuses on HIV and AIDS knowledge, attitudes, behavior, prevalence, misconception and stigma and higher-risk sexual behavior. However, the demand for AIS is less than that for DHS simply because the DHS was also tailored to collect the same HIV as a subset of the bigger DHS and majority of countries requested DHS rather than AIS. The target of DHS is not exclusively to collect and catalogue. Rather, evaluating valuable statistics on disease, fertility and nutrition is critical to solving global issues. Biological markers and GPS data are often collected with the survey questionnaires for maximum accuracy and complementing the survey results.

The sample design for both DHS and AIS is a two-stage cluster sampling which is representative at the national and sub-national level for both urban and rural residences. Depending on the country, the first stage is a minimum of 60 sample points selected in both the urban and rural domains for a total of 120 clusters. The second stage includes a section of an average of 25 households, all women aged 15–49 years and men aged 15–59 years are eligible to participate in the HIV testing.

Through an online platform called STATcompiler, DHS are viewable in scatter plots, charts, maps and statistical tables, all arranged by indicators and year. The DHS data available at the STATcompiler database is stratified at the national level, first and second administrative sub-divisions for each country’s DHS on several indicators. Equally, Geographic Information System (GIS) data linked with DHS indicators at the Spatial Data Repository are calibrated at the national, admin 1 and 2 subdivisions. However, our analysis was limited at the national and the first administrative subdivisions for accurate analysis and comparison. The data at the Spatial Data Repository and their corresponding shapefiles format were used in ArcGIS software 10.4 to describe the spatial distribution of HIV in the MRU countries. Geostatistical analysis was used to determine clusters and hot spots of HIV and significant results. The data at the STATcompiler was used for statistical analysis and predicting our sets of comprehensive correct knowledge (CCK) about HIV/AIDS that will be accompanied by a scale-up of HIV testing uptake in the MRU nations using LASSO regression. The DHS Spatial Data Repository uses Geographic Positioning System (GPS) data and GIS technology to link survey clusters, indicators and their attributes in shapefiles to foster within and between countries’ comparison over space. To attain confidentiality of the respondents, the centers of survey clusters are masked to prevent possible identification of participants by DHS data users. STATcompiler and Spatial Data Repository are funded by the United States Agency for International Development (USAID), Produced by ICF International, for easy use of DHS data.

The design for DHS is a two-stage sample: clusters and households are sampling units, and usually, all adults aged 15–59 years in selected households are eligible to participate. However, the majority of the surveys were done among respondents aged 15-49 years, and depending on the survey, questionnaires for men and biomarkers are administered only in a subsample of the selected households for data collection. Through a face-to-face interview, a standardized questionnaire is administered to consenting respondents by trained interviewers. The resulting data are then calculated and recalculated and presented in a different format for easy usage by researchers, policymakers and programme managers.

**Spatial distribution of HIV hot spots and spatial stratified heterogeneity (SSH) analyses**

The Incremental Spatial Autocorrelation tool runs the Spatial Autocorrelation (Global Moran’s I) for a series of increasing distances, measuring the intensities of spatial clustering for each distance. The intensity of clustering is defined by the Z-Score returned. Basically, as the distance increases, so does the Z- Score, indicating intensification of clustering. This tool measures spatial autocorrelation for a series of distances and optionally creates a line graph of those distances and their corresponding z-scores. Z-scores reflect the intensity of spatial clustering, and statistically significant peak z-scores indicate distances where spatial processes promoting clustering are pronounced (Figure S1 (A, B & C)). These peak distances are often appropriate values to use for tools with a Distance Band or Distance Radius Parameter in the Getis Ord Gi* tool.

Geodetector statistical software is used to describe Spatial Stratified Heterogeneity (SSH) and its attributes for two or more variables in geographic space. It has four functions; a) the risk detector maps response variable in strata, b) the factor detector q-statistic measures the degree of SH of a variable, and the determinant power of an explanatory variable, c) the ecological detector identifies the differences of the impacts between two explanatory variable, d) the interaction detector reveals whether the risk factors have an interactive influence on the response variable.

**S1.2 HIV temporal Changes**

The HIV temporal changes in the Mano River Union was determined by subtracting the HIV prevalence of the second DHS in our analysis (2011–2015) from the HIV prevalence of the first DHS (2005–2010) , and subtracting the HIV prevalence of the third DHS (2016–2020) from the HIV prevalence of the second DHS for each country. The HIV prevalence for each groups of DHS and the HIV change are represented in the bar chart map graph, while the temporal changes is represented in thematic maps.

**S1.3 Least absolute shrinkage and selection operator (LASSO)**

LASSO regression is a machine learning aimed at improving prediction. LASSO regression effectively select only the most important predictor variable for predicting an outcome by shrinking the regression coefficients associated with the least important predictor variable to zero. LASSO is similar to Ridge Regression. It works by introducing a bias term but instead of squaring the slope, the absolute value of the slope is added as a penalty term. Ridge Regression can reduce the slope close to zero (but not exactly zero). However LASSO regression can reduce the slope to be exactly equal to zero. LASSO regression can exclude useless variables from equations. This makes the final equation simpler and easier to interpret. LASSO regression helps reduce over-fitting and it’s particularly useful for future selection and can be useful if we have several independent variables that are useless.

Technically, LASSO regression involves two tuning parameters called alpha (α) and lambda (ʎ). During the model training, α=1 and lambda can be varied. The effect of alpha (α) on LASSO regression is similar to its effect on ridge regression. As alpha increases, the slope of the regression line reduced and becomes more horizontal. As alpha increases, the model becomes less sensitive to the variation of the independent variable. Lambda places a constraint on the maximum absolute value of the regression coefficients in the model and adds a penalty to non-zero regression coefficient in the model.

LASSO regression is a regularization method, especially an L1 regularization method, and is related to other regularization like Ridge regression and Elastic net regression. The purpose of regularization is to reduce variance of parameter estimate (ie. Regression coefficient), even if it comes at the expense of some additional bias; ultimately, this means finding the optimal level of model complexity. The regression analysis performed by LASSO is based on its inherent shrinkage parameter, which shrinks the magnitude of the independent variable’s coefficients according to its predictive power. Therefore, the coefficients of some variables may shrink to zero, allowing us to restrict the model to variables with non-zero coefficients.

The glmnet package is an amazing tool in machining learning using regression models like LASSO. The path of regularization is computed for the LASSO or elastic net penalty at a grid of values for the regularization parameter lambda. This wonderful package in R can also fit relaxed LASSO regression, generalized linear models for custom families, and multi-response linear regression models. The glmnet package has immense applications in regression analyses: GLM family function in glmnet describes how to fit custom generalized linear model (GLMs) with the elastic net penalty via the family argument, and the Relaxed LASSO describes how to fit relaxed LASSO regression model using the relaxed family of argument, and Regularized Cox Regression describes how to fit regularized cox models for survival data with glmnet.

The glmnet algorithms employ cyclical coordinate descent, which successively optimizes the objective function over each parameter with others fixed, and cycles repeatedly until convergence. The glmnet further makes use of the strong rules for efficient restriction of the active set. The core of glmnet is a set of FORTRAN subroutines, which makes for very fast execution. The code can handle sparse input-matrix formats, as well as range constraints on coefficient.

**Cross-validation**

There are other functions in the glmnet package like the CV-glmnet. K-fold cross-validation can be performed using the CV-glmnet function. CV-glmnet is one of the parameters of glmnet package.

How accurate is LASSO? LASSO model is designed with improved accuracy in predicting an outcome. In our LASSO regression model, the accuracy of the LASSO model was improved by the K-fold cross-validation of the model using the CV-glmnet. Cross-validation is a very necessary tool to evaluate the LASSO model for classification accuracy. In addition to that, the maximum area under the curve (AUC), also known as lambda.1se provides the most regularized model such that the cross-validated error is within one standard error of the minimum. To further test the reliability of our regression, the stability of our final model was tested by repeating the cross-validation 1000 times. The recurrence rate of a parameter that appears in the final models of these 1000 repetitions was used to assess the stability of this parameter. Only parameters with recurrence rate >80% were then considered robust parameters in the final model. Thus, the accuracy of our LASSO model was highly improved in validating our predictions.

| **Country** | **Survey Type** | **Survey**  **Year** | **Number of clusters** | **Number of Households** | **Urban** | **Response**  **Rate (%)** | **Rural** | **Response**  **Rate (%)** | **Sample**  **Size** | **Women** | **Response**  **Rate (%)** | **Men** | **Response**  **Rate (%)** |
| --- | --- | --- | --- | --- | --- | --- | --- | --- | --- | --- | --- | --- | --- |
| **Cote d’Ivoire** | **AIS** | **2005** | **247** | **4360** | **1885** | **94.2** | **2483** | **96.6** | **9686** | **5183** | **89.8** | **4503** | **87.5** |
| **Cote d’Ivoire** | **DHS** | **2012** | **351** | **9686** | **4013** | **97.2** | **5673** | **98.7** | **15737** | **10060** | **92.7** | **5677** | **90.5** |
| **Guinea** | **DHS** | **2005** | **297** | **6282** | **1718** | **98.1** | **4564** | **99.6** | **11128** | **7954** | **97.2** | **3174** | **94.5** |
| **Guinea** | **DHS** | **2012** | **300** | **7109** | **2503** | **98.9** | **4606** | **99.8** | **12924** | **9142** | **98** | **3782** | **96.7** |
| **Guinea** | **DHS** | **2018** | **401** | **7912** | **2701** | **98.7** | **5211** | **99.4** | **14991** | **10874** | **99** | **4117** | **97.2** |
| **Liberia** | **DHS** | **2007** | **298** | **6824** | **2606** | **95.8** | **4218** | **98.1** | **13101** | **7092** | **95.2** | **6009** | **92.8** |
| **Liberia** | **DHS** | **2013** | **322** | **9333** | **3450** | **99.5** | **5883** | **99.4** | **13357** | **9239** | **97.6** | **4118** | **95.4** |
| **Liberia** | **DHS** | **2019** | **325** | **9068** | **3321** | **97.6** | **5747** | **99** | **12314** | **8065** | **96.4** | **4249** | **93.9** |
| **Sierra Leone** | **DHS** | **2008** | **353** | **7284** | **2956** | **96.3** | **4328** | **98.5** | **10654** | **7374** | **94** | **3280** | **92.6** |
| **Sierra Leone** | **DHS** | **2013** | **435** | **12629** | **4569** | **98.8** | **8060** | **99.5** | **23920** | **16658** | **97.2** | **7262** | **96.4** |
| **Sierra Leone** | **DHS** | **2019** | **578** | **13399** | **4976** | **98.9** | **8423** | **98.3** | **20596** | **13399** | **98.5** | **7197** | **96.9** |

**Table S1. Sample sizes and response rate for different DHS in the MRU countries from 2005 to 2020^a^**

**^b^Source: DHS final reports**

**^a^Samples sizes for each country and DHS showing the differences in response rate between urban and rural residence, and between adult women and men aged 15-49 years.**

| **Table S2** |  |  |  | **LASSO regression model for comprehensive correct knowledge (CCK) about HIV/AIDS and HIV testing uptake for women aged 15-49 years** | | | | | | | | | | | | | | | | |  |  |  |  |  |  |
| --- | --- | --- | --- | --- | --- | --- | --- | --- | --- | --- | --- | --- | --- | --- | --- | --- | --- | --- | --- | --- | --- | --- | --- | --- | --- | --- |
| **Country** | **Region** | **Year** | **CCK1** | **CCK2** | **CCK3** | **CCK4** | **CCK5** | **CCK6** | **CCK7** | **CCK8** | **CCK9** | **CCK10** | **CCK11** | **CCK12** | **CCK13** | **CCK14** | **CCK15** | **CCK16** | **CCK17** | **CCK18** | **T1** | **T2** | **T3** | **T4** | **T5** | **T6** |
| **Cote d'Ivoire** | **Abidjan** | **2005** | **95.6** | **68.4** | **75.7** | **60.5** | **17.5** | **74.9** | **47.4** | **53.9** | **77.1** | **25.8** | **70.2** | **66.5** | **55.4** | **89.4** | **60.9** | **77.6** | **23.4** | **8.6** | **58.6** | **20.9** | **3** | **76.2** | **23.8** | **7.4** |
| **Cote d'Ivoire** | **Central** | **2005** | **93.7** | **77** | **81.3** | **71.8** | **20** | **67.9** | **39.9** | **42.1** | **63.4** | **22.6** | **70.5** | **45.5** | **40.8** | **83.5** | **44.6** | **62.9** | **28.8** | **5.8** | **29.3** | **8.2** | **1.5** | **90.3** | **9.7** | **2.1** |
| **Cote d'Ivoire** | **East Central** | **2005** | **93.3** | **67** | **80.7** | **64** | **12.7** | **68** | **31.8** | **38.2** | **58.4** | **14.8** | **67.5** | **37.4** | **32.7** | **76.7** | **32.4** | **49.3** | **36.9** | **7.1** | **26.1** | **6.4** | **1.3** | **92.3** | **7.7** | **2.3** |
| **Cote d'Ivoire** | **North Central** | **2005** | **84.6** | **41.8** | **46.5** | **34.1** | **10** | **43.4** | **36.1** | **41.8** | **41.2** | **16.7** | **44.5** | **24.4** | **19.9** | **81** | **38.1** | **41.5** | **38.8** | **7.1** | **19.5** | **5.9** | **0.1** | **94** | **6** | **2.3** |
| **Cote d'Ivoire** | **West Central** | **2005** | **88.5** | **67.8** | **78.3** | **65.5** | **16.6** | **57.1** | **34.6** | **49.3** | **52.8** | **18** | **63.5** | **33.6** | **26.6** | **84.5** | **40.2** | **64.4** | **39.4** | **12.8** | **27.4** | **7.1** | **1.8** | **91.1** | **8.9** | **2.7** |
| **Cote d'Ivoire** | **North** | **2005** | **66.1** | **23.8** | **26.2** | **23.5** | **7.5** | **22.3** | **18.2** | **17.4** | **19.5** | **8.2** | **22.5** | **9.3** | **8.5** | **79.6** | **28.4** | **37.6** | **36.2** | **9.8** | **6.9** | **0.7** | **0.1** | **99.3** | **0.7** | **0.2** |
| **Cote d'Ivoire** | **Northeast** | **2005** | **85.4** | **60.5** | **70.7** | **59.7** | **10.7** | **56.1** | **21.4** | **27.8** | **38.3** | **10.8** | **53.8** | **19.6** | **18.1** | **81.2** | **32.6** | **56** | **33.1** | **6.6** | **7.4** | **1.9** | **0.3** | **97.9** | **2.1** | **1.1** |
| **Cote d'Ivoire** | **Northwest** | **2005** | **67.8** | **40.8** | **44.8** | **38.7** | **18.2** | **45.2** | **39.8** | **38.1** | **28.6** | **21.8** | **42.4** | **26.7** | **18.3** | **76.2** | **22.3** | **22.9** | **32.1** | **1.9** | **5.1** | **0.5** | **0.6** | **98.9** | **1.1** | **0.2** |
| **Cote d'Ivoire** | **West** | **2005** | **80.2** | **48** | **59.3** | **44.3** | **11.4** | **37** | **42.8** | **37.1** | **38** | **16.7** | **62.4** | **26.1** | **25.1** | **78.2** | **38.5** | **29.3** | **55.8** | **7.3** | **8.7** | **2** | **2.3** | **95.8** | **4.2** | **1** |
| **Cote d'Ivoire** | **South** | **2005** | **97.2** | **61.8** | **74.8** | **55.6** | **15.9** | **72.4** | **40.7** | **49.6** | **66.4** | **22.6** | **67.7** | **49.7** | **42.9** | **80** | **46.9** | **61.3** | **32.4** | **11.4** | **38.3** | **15.3** | **1.1** | **83.6** | **16.4** | **4.9** |
| **Cote d'Ivoire** | **Southwest** | **2005** | **93.5** | **63.8** | **76.3** | **57** | **29.7** | **66.7** | **54.8** | **64.5** | **75.6** | **35** | **64** | **47.8** | **40.9** | **71.4** | **32.4** | **47.7** | **45.4** | **7.1** | **19.8** | **5.9** | **1.6** | **92.5** | **7.5** | **1.2** |
| **Cote d'Ivoire** | **Abidjan** | **2012** | **98.6** | **72.9** | **78.5** | **62.3** | **22.5** | **80.3** | **59.4** | **52.8** | **79.1** | **30.7** | **78** | **80.3** | **69.6** | **89.3** | **69.7** | **81.2** | **17.9** | **9.6** | **77.7** | **47** | **2.8** | **50.2** | **49.8** | **19.4** |
| **Cote d'Ivoire** | **Central** | **2012** | **98.6** | **58.6** | **64.1** | **47.5** | **10.6** | **71.5** | **36.3** | **48.3** | **70** | **17.7** | **81.2** | **71.6** | **64.5** | **76.8** | **40.4** | **56.3** | **20.7** | **4** | **60.6** | **36.6** | **3.1** | **60.3** | **39.7** | **17.4** |
| **Cote d'Ivoire** | **East Central** | **2012** | **95.3** | **55.2** | **66** | **47.5** | **12.4** | **71.2** | **37.1** | **42.5** | **65.1** | **18.5** | **70.9** | **71.6** | **62.9** | **78.9** | **53.4** | **65.7** | **18.2** | **7.4** | **71.7** | **44.8** | **2.2** | **53** | **47** | **15.2** |
| **Cote d'Ivoire** | **North Central** | **2012** | **94** | **59.2** | **65.4** | **47.7** | **16.4** | **70.2** | **59.7** | **49** | **77.6** | **28.6** | **65.3** | **65.4** | **50** | **84.4** | **57.3** | **73** | **17.9** | **4.4** | **62.4** | **40.6** | **0.5** | **58.9** | **41.1** | **15.9** |
| **Cote d'Ivoire** | **West Central** | **2012** | **88.6** | **46.9** | **58** | **39.6** | **11.3** | **44.5** | **42.4** | **47.5** | **58.1** | **17.6** | **56.8** | **48.4** | **36.2** | **84** | **59.8** | **71.7** | **34** | **10.7** | **51.8** | **32.3** | **3.1** | **64.6** | **35.4** | **11.9** |
| **Cote d'Ivoire** | **North** | **2012** | **86.9** | **64** | **69.2** | **59.7** | **14.5** | **59.7** | **31.3** | **45.5** | **47.4** | **17.2** | **65.3** | **41.1** | **38.7** | **95.3** | **44.9** | **82.2** | **13.6** | **2.3** | **55.8** | **32.1** | **4.2** | **63.7** | **36.3** | **13.3** |
| **Cote d'Ivoire** | **Northeast** | **2012** | **90.8** | **56.9** | **68.1** | **51.1** | **10** | **57** | **32.8** | **39.9** | **54.2** | **13.6** | **61.9** | **54.3** | **49.7** | **81.6** | **43.8** | **64** | **22.6** | **5.2** | **48.3** | **20.3** | **2** | **77.7** | **22.3** | **8.6** |
| **Cote d'Ivoire** | **Northwest** | **2012** | **88.3** | **55.5** | **64.2** | **48.6** | **6.4** | **39** | **25.1** | **38.3** | **35.1** | **8.7** | **62** | **37.1** | **32** | **72.9** | **20.8** | **38.6** | **38.8** | **4.9** | **41.7** | **15.7** | **2.2** | **82.1** | **17.9** | **6.5** |
| **Cote d'Ivoire** | **West** | **2012** | **92.1** | **55** | **62.6** | **45.6** | **9.7** | **50.9** | **37.8** | **40.5** | **59.3** | **14.4** | **57.8** | **39.4** | **33.3** | **70** | **39.4** | **51.8** | **38.9** | **6.3** | **51.6** | **20.8** | **2.5** | **76.7** | **23.3** | **8.4** |
| **Cote d'Ivoire** | **South** | **2012** | **95.4** | **53.1** | **57.7** | **40.5** | **10.8** | **63.6** | **45.3** | **41** | **67.3** | **16.9** | **68.8** | **70.6** | **59.1** | **74.7** | **50.4** | **62.4** | **37.2** | **11.6** | **71.1** | **39.8** | **4.8** | **55.4** | **44.6** | **15** |
| **Cote d'Ivoire** | **Southwest** | **2012** | **98.2** | **65.1** | **69.2** | **56.8** | **9.8** | **59.3** | **39.7** | **40.6** | **57.9** | **13.1** | **76.9** | **51.5** | **48.9** | **71.2** | **43.9** | **58.4** | **22** | **4.2** | **50** | **23.1** | **1.8** | **75.1** | **24.9** | **9.1** |
| **Guinea** | **Conakry** | **2005** | **98.9** | **74.6** | **82** | **66.4** | **23.2** | **56.5** | **49.4** | **69.7** | **65.9** | **27.8** | **50.9** | **19.7** | **14.5** | **61.3** | **14.9** | **29.5** | **50** | **3.2** | **37.7** | **5.5** | **1.4** | **93.1** | **6.9** | **2.7** |
| **Guinea** | **Bonke’** | **2005** | **98.2** | **67.3** | **75.3** | **60.8** | **13.3** | **54** | **50.6** | **67.9** | **52.6** | **16.4** | **38.3** | **18.4** | **16** | **45.3** | **19** | **17** | **51.3** | **0.9** | **10.4** | **1.9** | **0.3** | **97.9** | **2.1** | **0.8** |
| **Guinea** | **Faranah** | **2005** | **98.4** | **66.5** | **91.9** | **63.8** | **7.8** | **41.3** | **44.3** | **50** | **30.8** | **10.4** | **29.6** | **1.1** | **0.5** | **27.2** | **5.2** | **18.3** | **73.3** | **1** | **12.8** | **0.4** | **0** | **99.6** | **0.4** | **0** |
| **Guinea** | **Kankan** | **2005** | **99.4** | **81** | **93.7** | **78.1** | **9.2** | **34.4** | **56.4** | **80.2** | **44.9** | **10.6** | **46** | **1.9** | **1.5** | **39.3** | **4.5** | **25.6** | **68.4** | **0.4** | **9.7** | **0.3** | **0.3** | **99.4** | **0.6** | **0.2** |
| **Guinea** | **Kindia** | **2005** | **93.4** | **76.8** | **91.7** | **76.3** | **23.7** | **59.7** | **37.1** | **54.2** | **41** | **24.1** | **64.6** | **11.3** | **10.5** | **66.4** | **5.9** | **12.7** | **85.9** | **2.8** | **9.1** | **1.4** | **0** | **98.6** | **1.4** | **0.6** |
| **Guinea** | **Labe’** | **2005** | **91** | **63.6** | **84.9** | **61.4** | **9** | **43.3** | **31.6** | **61.3** | **49.8** | **10.7** | **46** | **11.8** | **10.1** | **59.9** | **4.2** | **24.5** | **76.9** | **1.3** | **10.5** | **2** | **0.4** | **97.5** | **2.5** | **1.3** |
| **Guinea** | **Mamou** | **2005** | **92.9** | **54** | **82.1** | **50.2** | **9.2** | **33.2** | **35.4** | **56.7** | **44.1** | **11.3** | **41.6** | **16.9** | **13** | **47.6** | **5.9** | **17.8** | **79.3** | **1.5** | **21.1** | **0.4** | **0.1** | **99.4** | **0.6** | **0.2** |
| **Guinea** | **N’Ze’re’kore** | **2005** | **99.8** | **71.7** | **94.8** | **69.9** | **13.8** | **49.7** | **41.2** | **58.5** | **40.8** | **15.3** | **66.9** | **1.1** | **1** | **32.6** | **6.1** | **17.2** | **82.8** | **1.7** | **24** | **2.5** | **0.4** | **97.1** | **2.9** | **1.5** |
| **Guinea** | **Conakry** | **2012** | **99.2** | **78.7** | **85.6** | **80.5** | **29.9** | **71.8** | **56.4** | **83.3** | **73.9** | **37.1** | **48.7** | **47.7** | **38.6** | **78.2** | **23.6** | **44.5** | **38.5** | **4** | **52.9** | **24.3** | **0.9** | **74.8** | **25.2** | **12.6** |
| **Guinea** | **Bonke’** | **2012** | **94.5** | **69.4** | **85.3** | **71** | **29** | **70** | **48.7** | **77.3** | **54.6** | **31.7** | **20.6** | **33.8** | **28.7** | **67** | **20.2** | **26.8** | **51.5** | **5.4** | **48** | **10** | **0.3** | **89.7** | **10.3** | **4.1** |
| **Guinea** | **Faranah** | **2012** | **92.7** | **72.8** | **85.7** | **64.1** | **13.9** | **57.2** | **53.7** | **78.9** | **49.7** | **16.6** | **47.6** | **15.9** | **12.2** | **35.2** | **8.1** | **15.4** | **66.5** | **1.1** | **19.1** | **4.7** | **0.2** | **95** | **5** | **1.4** |
| **Guinea** | **Kankan** | **2012** | **98.2** | **62.2** | **82.1** | **82.7** | **17.3** | **69.3** | **43.7** | **83.3** | **65.4** | **23.4** | **78.9** | **31** | **29.4** | **66.2** | **10.6** | **19.3** | **60.5** | **1.3** | **49.4** | **3.7** | **0.1** | **96.1** | **3.9** | **1.3** |
| **Guinea** | **Kindia** | **2012** | **94.4** | **68.1** | **78.8** | **69.2** | **17.2** | **40.5** | **50.3** | **71.5** | **56.3** | **18.4** | **45.8** | **33** | **30.7** | **54.3** | **12.2** | **21.2** | **65.2** | **1.5** | **25.3** | **8.5** | **0.6** | **90.9** | **9.1** | **3.7** |
| **Guinea** | **Labe’** | **2012** | **91.3** | **52.6** | **75.9** | **56.8** | **8.9** | **59.1** | **28.8** | **56.2** | **47.1** | **15** | **43** | **34.2** | **31.1** | **60.1** | **16.4** | **23.3** | **62** | **3** | **34.7** | **9.8** | **1** | **89.2** | **10.8** | **1.5** |
| **Guinea** | **Mamou** | **2012** | **94.4** | **60.1** | **84** | **80.2** | **17.2** | **57.6** | **56.1** | **69.8** | **57.4** | **25.3** | **72.6** | **22.2** | **14.3** | **61.1** | **23.9** | **31.9** | **56.1** | **2.8** | **14.9** | **5.7** | **0.4** | **93.9** | **6.1** | **2** |
| **Guinea** | **N’Ze’re’kore** | **2012** | **89.7** | **60.6** | **80.8** | **76.9** | **13.9** | **57.6** | **41.2** | **70.6** | **54.3** | **20.5** | **61.2** | **24.1** | **19.9** | **56** | **12.8** | **26.1** | **55.2** | **3** | **44.9** | **6.3** | **0.2** | **93.5** | **6.5** | **3.6** |
| **Guinea** | **Conakry** | **2018** | **92.3** | **70.1** | **78.5** | **65** | **36.8** | **69.3** | **63.9** | **77.8** | **70.1** | **44.8** | **54** | **55.2** | **40.1** | **NA^b^** | **27** | **NA^b^** | **NA^b^** | **NA^b^** | **58.8** | **31.8** | **1.1** | **67.1** | **32.9** | **17.8** |
| **Guinea** | **Bonke’** | **2018** | **71.2** | **47.1** | **60.5** | **44.9** | **12.2** | **27.6** | **45.5** | **59.6** | **45.6** | **13.8** | **38.9** | **28.9** | **20.7** | **NA^b^** | **12.5** | **NA^b^** | **NA^b^** | **NA^b^** | **36.3** | **15.1** | **0.8** | **84.1** | **15.9** | **6.6** |
| **Guinea** | **Faranah** | **2018** | **75.9** | **47.7** | **60.9** | **43.3** | **12.7** | **46** | **49.7** | **59** | **36.2** | **19.5** | **57** | **23.9** | **22.5** | **NA^b^** | **15.6** | **NA^b^** | **NA^b^** | **NA^b^** | **41.1** | **14.9** | **1** | **84** | **16** | **6.1** |
| **Guinea** | **Kankan** | **2018** | **83.9** | **50.9** | **70.9** | **47.6** | **12.4** | **49.3** | **34.4** | **59.4** | **54.4** | **20.3** | **53.5** | **42.2** | **38.9** | **NA^b^** | **26.6** | **NA^b^** | **NA^b^** | **NA^b^** | **42.8** | **16.6** | **0.3** | **83** | **17** | **10.9** |
| **Guinea** | **Kindia** | **2018** | **86.5** | **67.9** | **76.1** | **64.9** | **32.2** | **62.3** | **59.9** | **69.7** | **60.4** | **35.6** | **45.1** | **43.9** | **36.5** | **NA^b^** | **11.8** | **NA^b^** | **NA^b^** | **NA^b^** | **46.3** | **17.7** | **0.5** | **81.7** | **18.3** | **10.3** |
| **Guinea** | **Labe’** | **2018** | **62.1** | **42.5** | **48.3** | **38.2** | **6.5** | **30.3** | **25.8** | **42.9** | **24.9** | **7.4** | **30.6** | **25.3** | **19.6** | **NA^b^** | **10.8** | **NA^b^** | **NA^b^** | **NA^b^** | **18.4** | **5.4** | **0.4** | **94.1** | **5.9** | **2.8** |
| **Guinea** | **Mamou** | **2018** | **86.2** | **44.4** | **53.5** | **41** | **15.4** | **44.9** | **40.2** | **58.4** | **35** | **18.9** | **45.9** | **34.9** | **32.4** | **NA^b^** | **10.1** | **NA^b^** | **NA^b^** | **NA^b^** | **30.2** | **14.7** | **0.3** | **84.9** | **15.1** | **5.8** |
| **Guinea** | **N’Ze’re’kore** | **2018** | **79.2** | **42.3** | **57.2** | **38.7** | **19.9** | **42.3** | **61.8** | **73.7** | **59.5** | **29.8** | **52.9** | **24.1** | **21.1** | **NA^b^** | **23.3** | **NA^b^** | **NA^b^** | **NA^b^** | **45.8** | **14.6** | **1.3** | **84.1** | **15.9** | **5.8** |
| **Liberia** | **Monrovia** | **2007** | **97.9** | **60.6** | **67.7** | **55.3** | **29.1** | **75** | **64.9** | **74.7** | **71.8** | **47.3** | **70.2** | **15.4** | **13.9** | **57.7** | **46.9** | **46.9** | **65.4** | **14.7** | **35.8** | **5** | **0.9** | **94.1** | **5.9** | **2.6** |
| **Liberia** | **North Western** | **2007** | **91** | **24.4** | **24.4** | **16.6** | **7.3** | **47.2** | **51.8** | **61.9** | **51.4** | **32.2** | **30.8** | **4.5** | **3.4** | **55.1** | **31.6** | **30.6** | **72.2** | **18** | **13.8** | **2.4** | **0.2** | **97.4** | **2.6** | **1.6** |
| **Liberia** | **South Central** | **2007** | **92.2** | **55.2** | **71.7** | **48.5** | **19.9** | **67.9** | **49.7** | **68** | **66.7** | **34.7** | **69.1** | **17** | **15.3** | **56.6** | **38.5** | **34.4** | **68.2** | **11.9** | **19.4** | **3.7** | **1.4** | **94.9** | **5.1** | **2** |
| **Liberia** | **South Eastern A** | **2007** | **82.3** | **38.9** | **39.6** | **27.7** | **9.6** | **52.1** | **44.5** | **56.1** | **54.6** | **27** | **48.5** | **5.1** | **4.6** | **54** | **41.1** | **36.3** | **67.2** | **9.4** | **11.8** | **1.9** | **0.4** | **97.6** | **2.4** | **0.7** |
| **Liberia** | **South Eastern B** | **2007** | **86.4** | **26.8** | **43.1** | **22.7** | **15.7** | **52.8** | **50.2** | **54.6** | **56.3** | **40** | **47.6** | **5.8** | **5.3** | **60.3** | **50.1** | **48.7** | **54.1** | **16.8** | **12.3** | **0.8** | **0.3** | **98.9** | **1.1** | **0.3** |
| **Liberia** | **North Central** | **2007** | **80.9** | **48.2** | **55.9** | **44.4** | **14.7** | **59** | **41.5** | **63.1** | **57.2** | **27.9** | **60.2** | **15.7** | **13.8** | **37.6** | **21.4** | **28.1** | **86.8** | **8.5** | **20.6** | **2.1** | **0.9** | **97.1** | **2.9** | **1** |
| **Liberia** | **Monrovia** | **2013** | **99.7** | **81.4** | **84.5** | **75** | **50** | **85.4** | **74** | **84.4** | **85.5** | **60.9** | **76.7** | **68.4** | **59.4** | **78.4** | **54.8** | **60.8** | **30.9** | **7** | **85.3** | **50.8** | **4.3** | **44.9** | **55.1** | **19.7** |
| **Liberia** | **North Western** | **2013** | **98.2** | **72.5** | **72.7** | **61.7** | **29.6** | **68.5** | **57.5** | **74.2** | **68** | **38.9** | **67.6** | **45.1** | **40.5** | **59.9** | **38.8** | **41.2** | **52.3** | **7.1** | **72.7** | **44.2** | **6.9** | **48.9** | **51.1** | **22.6** |
| **Liberia** | **South Central** | **2013** | **99.3** | **79.1** | **82.6** | **72.8** | **45.1** | **78.9** | **71.9** | **83** | **80.7** | **54.3** | **75.3** | **64.6** | **57.6** | **76.2** | **51.7** | **57.7** | **33.4** | **7.1** | **80.9** | **47.5** | **4.4** | **48.1** | **51.9** | **19** |
| **Liberia** | **South Eastern A** | **2013** | **94** | **70.6** | **71.2** | **60.8** | **29.3** | **63.4** | **61.8** | **76.9** | **72.1** | **39.5** | **67** | **46.5** | **41** | **72.2** | **42.9** | **51.9** | **35.1** | **4** | **75** | **49.6** | **7.7** | **42.8** | **57.2** | **24.6** |
| **Liberia** | **South Eastern B** | **2013** | **91.3** | **64.9** | **73.2** | **59.1** | **29.8** | **60.2** | **63.8** | **75.7** | **67.8** | **37.2** | **60.3** | **48.1** | **40.4** | **71.6** | **38.1** | **43.4** | **46.5** | **7.7** | **55.3** | **29.9** | **8.4** | **61.7** | **38.3** | **16.1** |
| **Liberia** | **North Central** | **2013** | **95.2** | **71.2** | **77** | **64.2** | **25.3** | **65.8** | **59.5** | **73.1** | **67.1** | **33.8** | **66.7** | **53.5** | **47.4** | **55.6** | **32.7** | **33.9** | **58.1** | **5.4** | **73.1** | **43.2** | **7** | **49.8** | **50.2** | **17.7** |
| **Liberia** | **Monrovia** | **2019** | **95.5** | **71.5** | **75.7** | **65.4** | **40** | **84.3** | **68.9** | **78.5** | **74.5** | **52.9** | **69** | **59** | **52.3** | **NA^b^** | **32.9** | **NA^b^** | **NA^b^** | **NA^b^** | **70.5** | **53** | **3.9** | **43.1** | **56.9** | **21.5** |
| **Liberia** | **North Western** | **2019** | **92.2** | **65.9** | **77.4** | **59** | **27.7** | **63.5** | **62.5** | **77.6** | **65.8** | **39.4** | **68** | **42.9** | **39.9** | **NA^b^** | **41.3** | **NA^b^** | **NA^b^** | **NA^b^** | **73.8** | **50.7** | **3.2** | **46.2** | **53.8** | **24.3** |
| **Liberia** | **South Central** | **2019** | **94.9** | **72.6** | **76.3** | **66** | **38.1** | **79.4** | **66.5** | **77.6** | **73.3** | **49.6** | **68.2** | **57** | **51.3** | **NA^b^** | **34.7** | **NA^b^** | **NA^b^** | **NA^b^** | **70.7** | **51.3** | **4.1** | **44.6** | **55.4** | **21.2** |
| **Liberia** | **South Eastern A** | **2019** | **99.4** | **82.9** | **91.7** | **79.4** | **41** | **71.7** | **70.2** | **81** | **74** | **46.3** | **78.1** | **55.8** | **54** | **NA^b^** | **40.4** | **NA^b^** | **NA^b^** | **NA^b^** | **82.5** | **56.1** | **2.4** | **41.5** | **58.5** | **20.4** |
| **Liberia** | **South Eastern B** | **2019** | **97.5** | **75.5** | **83.7** | **69.1** | **29.6** | **71.2** | **63.8** | **72.8** | **65.2** | **39.9** | **71.6** | **65.5** | **60.3** | **NA^b^** | **38.8** | **NA^b^** | **NA^b^** | **NA^b^** | **81.2** | **50.8** | **2.4** | **46.7** | **53.3** | **21.1** |
| **Liberia** | **North Central** | **2019** | **92.9** | **71.6** | **80.4** | **66.1** | **26.4** | **61.6** | **55.4** | **67.2** | **60.4** | **32** | **64.4** | **48.8** | **43.1** | **NA^b^** | **32.9** | **NA^b^** | **NA^b^** | **NA^b^** | **71.4** | **47.3** | **5.7** | **47** | **53** | **23.3** |
| **Sierra Leone** | **Eastern** | **2008** | **69.6** | **36.9** | **38.8** | **28.4** | **12.7** | **28.1** | **34.8** | **44.2** | **38.1** | **19.8** | **39.6** | **12.1** | **10.9** | **47.4** | **23** | **28.3** | **69.5** | **7.6** | **21.5** | **5.6** | **2.8** | **91.6** | **8.4** | **2.5** |
| **Sierra Leone** | **Northern** | **2008** | **58.6** | **37.7** | **41.5** | **33.1** | **9.4** | **26.4** | **27.2** | **28.4** | **26.5** | **13.2** | **35.9** | **10.6** | **8.6** | **37.1** | **12.2** | **20.4** | **74** | **3.6** | **17.2** | **4.1** | **2.8** | **93.1** | **6.9** | **1.5** |
| **Sierra Leone** | **Southern** | **2008** | **65.3** | **34.4** | **40.7** | **31.1** | **9.5** | **31.7** | **23.3** | **35.4** | **30** | **14.7** | **39.9** | **10** | **9.6** | **52.7** | **18.7** | **25.6** | **68** | **6.6** | **26.5** | **7.6** | **3.2** | **89.2** | **10.8** | **4.2** |
| **Sierra Leone** | **Western** | **2008** | **94.9** | **68.8** | **77** | **62.9** | **29** | **73.3** | **53.1** | **67.9** | **60.8** | **37.5** | **70.9** | **27.3** | **25.2** | **62.5** | **28.9** | **48.5** | **35.7** | **4.4** | **58.5** | **25.5** | **4.6** | **70** | **30** | **10.6** |
| **Sierra Leone** | **Eastern** | **2013** | **93.5** | **58.1** | **66.8** | **54** | **20.3** | **51.7** | **55.9** | **66.7** | **51.9** | **28.5** | **76.1** | **58.2** | **56.2** | **68.9** | **43.7** | **50.8** | **53.7** | **7.2** | **68.7** | **43** | **9.5** | **47.5** | **52.5** | **13.7** |
| **Sierra Leone** | **Northern** | **2013** | **92.1** | **65.5** | **74** | **61.2** | **21.8** | **56.5** | **53.7** | **52.5** | **48.7** | **26.9** | **65.2** | **47.6** | **42.1** | **71.5** | **41.7** | **40.5** | **44.9** | **7.9** | **67.4** | **35** | **10.9** | **54.1** | **45.9** | **12.6** |
| **Sierra Leone** | **Southern** | **2013** | **98.7** | **82.4** | **86.6** | **77.2** | **37.5** | **74.7** | **63.5** | **71.2** | **66.1** | **42.2** | **77.5** | **65.5** | **59.7** | **78.6** | **49.9** | **59.7** | **26.6** | **3.3** | **79.5** | **42.1** | **15.1** | **42.8** | **57.2** | **15.9** |
| **Sierra Leone** | **Western** | **2013** | **NA^b^** | **NA^b^** | **NA^b^** | **NA^b^** | **NA^b^** | **NA^b^** | **NA^b^** | **NA^b^** | **NA^b^** | **NA^b^** | **NA^b^** | **NA^b^** | **NA^b^** | **NA^b^** | **NA^b^** | **NA^b^** | **NA^b^** | **NA^b^** | **NA^b^** | **NA^b^** | **NA^b^** | **NA^b^** | **NA^b^** | **NA^b^** |
| **Sierra Leone** | **Eastern** | **2019** | **94.6** | **76.1** | **79.1** | **68.5** | **24.3** | **58.3** | **70.4** | **78** | **53.2** | **28.7** | **67.4** | **51.4** | **49.2** | **NA^b^** | **23.6** | **NA^b^** | **NA^b^** | **NA^b^** | **79.3** | **40.5** | **13.3** | **46.2** | **53.8** | **19** |
| **Sierra Leone** | **Northern** | **2019** | **91.8** | **81.2** | **86** | **79.4** | **27.5** | **60.3** | **66** | **81.7** | **47.9** | **29.9** | **68.4** | **54.8** | **52.6** | **NA^b^** | **22.9** | **NA^b^** | **NA^b^** | **NA^b^** | **78.8** | **43** | **10** | **47** | **53** | **14.5** |
| **Sierra Leone** | **Southern** | **2019** | **88** | **69.7** | **74.6** | **64.8** | **32.7** | **60.5** | **59** | **69.9** | **53.3** | **35.8** | **69.6** | **49.9** | **49** | **NA^b^** | **21.4** | **NA^b^** | **NA^b^** | **NA^b^** | **68.4** | **40.7** | **10.1** | **49.1** | **50.9** | **16.8** |
| **Sierra Leone** | **Western** | **2019** | **98.9** | **70.6** | **86.1** | **67.8** | **31.5** | **82.8** | **77.9** | **87.7** | **62.1** | **46.5** | **78.9** | **69** | **64.1** | **NA^b^** | **29.8** | **NA^b^** | **NA^b^** | **NA^b^** | **86.6** | **52.2** | **13.1** | **34.7** | **65.3** | **28.4** |
| **Sierra Leone** | **North Western** | **2019** | **94.2** | **69.3** | **79.3** | **64.2** | **23.5** | **52.3** | **65.1** | **74.4** | **53.4** | **27.6** | **76.8** | **44.3** | **43.6** | NA**^b^** | **7.8** | NA**^b^** | NA^b^ | NA^b^ | **80.9** | **39.2** | **17** | **43.7** | **56.3** | **15.4** |

**^a^ICF, 2015. The DHS Program STATcompiler. Funded by USAID. http://www.statcompiler.com. December 29 2021**

**^b^NA = Not applicable; MTCT = Mother to child transmission**

**CCK1 =Women who have heard of HIV or AIDS**

**CCK2 =Knowledge of HIV prevention methods- use of condom**

**CCK3 =Knowledge of HIV prevention methods- one partner only**

**CCK4 =Knowledge of HIV prevention methods composite of 2 components**

**CCK5 =Comprehensive correct knowledge about AIDS**

**CCK6=No incorrect beliefs about AIDS- a healthy looking person can have the AIDS virus**

**CCK7 =No incorrect beliefs about AIDS- AIDS cannot be transmitted by mosquito bites**

**CCK8=No incorrect beliefs about AIDS- AIDS cannot be transmitted by supernatural means**

**CCK9 =No incorrect beliefs about AIDS- cannot become infected by sharing food with someone who has AIDS**

**CCK10 =No incorrect beliefs about AIDS- composite of 3 components**

**CCK11 =Knowledge of MTCT- Through breast feeding**

**CCK12 =prevention Knowledge of MTCT- can be prevented by mother taking special drugs during pregnancy**

**CCK13 =Knowledge of prevention of mother to child transmission of HIV**

**CCK14 =Accepting attitudes -willing to care for family member sick with AIDS**

**CCK15 =Accepting attitudes -would buy fresh vegetables from a shopkeeper with AIDS**

**CCK16 =Accepting attitudes – Female teacher who is HIV+ but not sick should be allowed to continue teaching in school**

**CCK17 =Accepting attitudes – Not secretive about family member's HIV status**

**CCK18 =Accepting attitudes towards those living with HIV- composites of 4 components**

**T1 =Women who know where to get an HIV test**

**T2 =Women ever tested for HIV and received test results**

**T3 =Women ever tested for HIV and did not receive test results**

**T4 =women never tested for HIV**

**T5 =Women ever receiving an HIV test**

**T6 =Women receiving an HIV test and receiving test results in the last 12 months before the survey.**

| **Table S3** |  |  |  |  | **LASSO regression model for comprehensive correct knowledge (CCK) about HIV/AIDS and HIV testing uptake for men aged 15-49 years** | | | | | | | | | | | | | | | | |  |  |  |  |  |
| --- | --- | --- | --- | --- | --- | --- | --- | --- | --- | --- | --- | --- | --- | --- | --- | --- | --- | --- | --- | --- | --- | --- | --- | --- | --- | --- |
| **Country** | **Region** | **Year** | **CCK1** | **CCK2** | **CCK3** | **CCK4** | **CCK5** | **CCK6** | **CCK7** | **CCK8** | **CCK9** | **CCK10** | **CCK11** | **CCK12** | **CCK13** | **CCK14** | **CCK15** | **CCK16** | **CCK17** | **CCK18** | **T1** | **T2** | **T3** | **T4** | **T5** | **T6** |
| **Cote d'Ivoire** | **Abidjan** | **2005** | **99.7** | **80.2** | **83** | **71.8** | **35.2** | **90.1** | **61.9** | **70.7** | **82.8** | **44.7** | **58.8** | **60.6** | **39.3** | **91.5** | **65.3** | **80.4** | **49.6** | **30.4** | **68.5** | **NA** | **NA** | **83.5** | **16.5** | **6** |
| **Cote d'Ivoire** | **Central** | **2005** | **97.5** | **83.2** | **87.4** | **78.3** | **30** | **85** | **50.5** | **66.2** | **79.3** | **35.6** | **70.9** | **51.1** | **39.5** | **83.2** | **54.6** | **70.2** | **53.3** | **22.4** | **56.1** | **NA** | **NA** | **90.6** | **9.4** | **2.9** |
| **Cote d'Ivoire** | **East Central** | **2005** | **97.7** | **74.1** | **77.6** | **68.3** | **20.7** | **79.3** | **41.1** | **53** | **65.6** | **26.6** | **60** | **39.4** | **25.2** | **83.4** | **40.6** | **57.8** | **52.4** | **14** | **51.1** | **NA** | **NA** | **91.5** | **8.5** | **2.9** |
| **Cote d'Ivoire** | **North Central** | **2005** | **89.5** | **58.4** | **57.7** | **47.9** | **16.5** | **66.1** | **44.5** | **53.1** | **58.2** | **27.3** | **49.9** | **25.1** | **19.5** | **86.7** | **52.3** | **57.9** | **42.7** | **16.4** | **39.3** | **NA** | **NA** | **94.3** | **5.7** | **2** |
| **Cote d'Ivoire** | **West Central** | **2005** | **98.2** | **78.1** | **86.4** | **73** | **24.1** | **75.7** | **45.5** | **59.8** | **63.6** | **28.1** | **67** | **31.5** | **21.7** | **74.4** | **44.6** | **61.9** | **60.3** | **21.9** | **48.2** | **NA** | **NA** | **95.6** | **4.4** | **0.6** |
| **Cote d'Ivoire** | **North** | **2005** | **75.7** | **47** | **44.4** | **42.8** | **15.9** | **45.5** | **31.6** | **36.7** | **34.3** | **19.5** | **27.5** | **8.2** | **7.2** | **84.7** | **32.9** | **43.1** | **40.8** | **11.1** | **9.7** | **NA** | **NA** | **99** | **1** | **0.2** |
| **Cote d'Ivoire** | **Northeast** | **2005** | **94.6** | **77.9** | **77.6** | **71.7** | **15.2** | **65.9** | **31.6** | **47.1** | **56.4** | **16.8** | **55.9** | **21.8** | **18.2** | **85.9** | **38.1** | **52.1** | **47.1** | **13.4** | **28.6** | **NA** | **NA** | **96.1** | **3.9** | **0.4** |
| **Cote d'Ivoire** | **Northwest** | **2005** | **72.7** | **47** | **47.8** | **41** | **9.6** | **47** | **33.1** | **40** | **36.6** | **16.6** | **52.5** | **22.6** | **19.8** | **81.5** | **35.8** | **39.3** | **53.2** | **8.9** | **17.7** | **NA** | **NA** | **96.4** | **3.6** | **0.6** |
| **Cote d'Ivoire** | **West** | **2005** | **83.8** | **68.1** | **74.3** | **65.1** | **11.2** | **50.7** | **29.1** | **50.3** | **32.5** | **13.8** | **55.6** | **19.7** | **17** | **80** | **34.9** | **51.3** | **80.1** | **18.3** | **11.5** | **NA** | **NA** | **96.9** | **3.1** | **1** |
| **Cote d'Ivoire** | **South** | **2005** | **98.1** | **80.9** | **85** | **71.9** | **31** | **82.2** | **54.1** | **69.1** | **76.2** | **36.5** | **61.2** | **54.3** | **35.7** | **90.5** | **57.8** | **65.6** | **47** | **20.8** | **58.5** | **NA** | **NA** | **86.6** | **13.4** | **4.9** |
| **Cote d'Ivoire** | **Southwest** | **2005** | **98.5** | **72.8** | **79.5** | **66.5** | **24.2** | **68.6** | **55.4** | **65.8** | **73.9** | **30.3** | **55.2** | **37.7** | **29.4** | **78.5** | **43.4** | **54.9** | **54.3** | **19.6** | **28.9** | **NA** | **NA** | **95** | **5** | **1** |
| **Cote d'Ivoire** | **Abidjan** | **2012** | **99.4** | **84.7** | **84.6** | **76** | **36.8** | **88.9** | **61.6** | **69.7** | **83.9** | **44.4** | **58.6** | **70.2** | **50.3** | **90.6** | **71.6** | **77.9** | **38.5** | **24** | **74.3** | **33.3** | **2.1** | **64.6** | **35.4** | **14.6** |
| **Cote d'Ivoire** | **Central** | **2012** | **99.2** | **87.9** | **86.3** | **81** | **41.1** | **81** | **57.9** | **68.8** | **52.9** | **43.5** | **72.9** | **36.1** | **30.2** | **80.1** | **25.9** | **67.7** | **27.3** | **6.2** | **56.8** | **21.6** | **0.4** | **78** | **22** | **5.3** |
| **Cote d'Ivoire** | **East Central** | **2012** | **98.2** | **82.6** | **83.6** | **73.5** | **25.2** | **86** | **47.2** | **57.5** | **76.2** | **28.5** | **65.5** | **68.9** | **51.4** | **88** | **57** | **72.9** | **30.5** | **12.9** | **69.7** | **27.2** | **3.5** | **69.3** | **30.7** | **11** |
| **Cote d'Ivoire** | **North Central** | **2012** | **99.6** | **79.6** | **83.9** | **71.6** | **28.9** | **84.5** | **48.1** | **72.2** | **76.6** | **36.9** | **59.2** | **58** | **41** | **83.4** | **51.6** | **68.8** | **28** | **12** | **67.8** | **24.7** | **3.2** | **72.1** | **27.9** | **11.8** |
| **Cote d'Ivoire** | **West Central** | **2012** | **96.9** | **76.3** | **70.9** | **60.3** | **12.4** | **65.3** | **34** | **57.1** | **64.8** | **19.7** | **61.6** | **47.7** | **38.8** | **90.1** | **50.7** | **55.3** | **34.4** | **11.8** | **52.5** | **17.9** | **2.5** | **79.6** | **20.4** | **6.1** |
| **Cote d'Ivoire** | **North** | **2012** | **88.5** | **68** | **66.8** | **58.1** | **20.3** | **56.4** | **36.5** | **60.5** | **56.4** | **24.7** | **49.9** | **39.7** | **29.9** | **76** | **45.5** | **69.8** | **17.2** | **6.7** | **42.5** | **18.9** | **3.1** | **78** | **22** | **11.1** |
| **Cote d'Ivoire** | **Northeast** | **2012** | **99.2** | **85** | **88.4** | **80.2** | **20.5** | **89.8** | **28.6** | **60.5** | **66** | **20.7** | **77** | **66.3** | **55.1** | **87.8** | **53.2** | **64.7** | **42.8** | **17.5** | **56.2** | **18.2** | **2.6** | **79.2** | **20.8** | **7.7** |
| **Cote d'Ivoire** | **Northwest** | **2012** | **92.4** | **69.8** | **79.4** | **63.4** | **11.6** | **71.1** | **22.5** | **49.1** | **44.9** | **13.4** | **59.2** | **35.6** | **29.8** | **84.3** | **31.2** | **45.3** | **41.2** | **8.5** | **34** | **7.4** | **1.8** | **90.8** | **9.2** | **1.2** |
| **Cote d'Ivoire** | **West** | **2012** | **98.7** | **81.2** | **78.1** | **69.6** | **18.3** | **62.5** | **39.8** | **63.1** | **66.7** | **23.2** | **64.8** | **44.5** | **36** | **73.2** | **48.5** | **50.1** | **54.6** | **17** | **53** | **13.3** | **2.9** | **83.8** | **16.2** | **4.1** |
| **Cote d'Ivoire** | **South** | **2012** | **98.2** | **80.1** | **74.9** | **63.2** | **24.4** | **80.9** | **47.5** | **58.1** | **76.1** | **29.9** | **61.3** | **64.8** | **48.1** | **87.1** | **65.2** | **69.6** | **35.7** | **19.5** | **63.8** | **27.7** | **0.6** | **71.6** | **28.4** | **12.5** |
| **Cote d'Ivoire** | **Southwest** | **2012** | **99.3** | **72.7** | **76.5** | **61** | **14.3** | **57** | **36.8** | **52.4** | **67.1** | **19.5** | **63** | **45.2** | **33.8** | **88.1** | **44.8** | **53.2** | **33.6** | **11.7** | **54.7** | **18.1** | **1.8** | **80.1** | **19.9** | **6.9** |
| **Guinea** | **Conakry** | **2005** | **99.8** | **81.9** | **86** | **73.6** | **27.6** | **81** | **43.7** | **NA** | **65.5** | **34.4** | **56** | **21** | **13.9** | **72.2** | **24.5** | **43.5** | **69.9** | **11.2** | **56.4** | **12.1** | **1** | **86.9** | **13.1** | **5.3** |
| **Guinea** | **Bonke’** | **2005** | **99** | **83** | **93.5** | **80.9** | **13.2** | **76.6** | **24** | **NA** | **40.6** | **14.8** | **71.6** | **22.9** | **20.9** | **62.7** | **11.4** | **23.5** | **83.9** | **4.8** | **45.2** | **7.8** | **1.2** | **91.1** | **8.9** | **3.8** |
| **Guinea** | **Faranah** | **2005** | **100** | **79.3** | **91.4** | **77.4** | **18.4** | **75.3** | **27.6** | **NA** | **56.3** | **19.5** | **69.1** | **3.9** | **3.7** | **68.7** | **20.4** | **32.4** | **90.3** | **11** | **48.9** | **4.6** | **0.4** | **95** | **5** | **0.8** |
| **Guinea** | **Kankan** | **2005** | **98.7** | **82.1** | **91.9** | **79.7** | **16.5** | **63.6** | **30.7** | **NA** | **45.4** | **18.1** | **81.3** | **21.1** | **19.3** | **47.7** | **14.1** | **21.3** | **74.4** | **4.6** | **42.9** | **2.4** | **0** | **97.6** | **2.4** | **1.6** |
| **Guinea** | **Kindia** | **2005** | **95.3** | **79.3** | **87.2** | **74.1** | **18.7** | **67.4** | **30.9** | **NA** | **37.9** | **21.2** | **62** | **23.7** | **20.6** | **77.9** | **18.5** | **22.4** | **66.2** | **2.8** | **44.3** | **3.2** | **0** | **96.8** | **3.2** | **1** |
| **Guinea** | **Labe’** | **2005** | **98.5** | **75.9** | **94.4** | **73.5** | **27.3** | **66.5** | **41.1** | **NA** | **64.2** | **31.1** | **78.9** | **14.8** | **10.9** | **80.1** | **7.5** | **33.4** | **78** | **5.3** | **13.8** | **0.3** | **0** | **99.7** | **0.3** | **0** |
| **Guinea** | **Mamou** | **2005** | **96** | **82.5** | **94** | **81.5** | **17.7** | **55.4** | **27.4** | **NA** | **43** | **17.7** | **62.3** | **13.6** | **11.2** | **39.2** | **11.3** | **21.6** | **89.4** | **3.9** | **36.9** | **4.5** | **0** | **95.5** | **4.5** | **1.1** |
| **Guinea** | **N’Ze’re’kore** | **2005** | **100** | **85.9** | **98.9** | **85.4** | **18.4** | **57.2** | **41.6** | **NA** | **40.3** | **19** | **65.7** | **6.4** | **5.7** | **86.7** | **31.5** | **47.3** | **63.1** | **3.9** | **41.7** | **5** | **1.3** | **93.7** | **6.3** | **3.6** |
| **Guinea** | **Conakry** | **2012** | **99.3** | **87.7** | **89.1** | **80.5** | **40.2** | **72.1** | **67.6** | **88.9** | **78.2** | **46.5** | **48.7** | **44.4** | **28.5** | **89.4** | **39.1** | **54.5** | **56.1** | **15.4** | **63.7** | **20.9** | **1** | **78.1** | **21.9** | **9** |
| **Guinea** | **Bonke’** | **2012** | **92** | **74.6** | **83.2** | **71** | **45.8** | **61.3** | **71.2** | **83** | **80.3** | **49** | **20.6** | **18.8** | **9.5** | **80.7** | **12.2** | **48.4** | **82.2** | **6.7** | **27.8** | **4.2** | **0.4** | **95.5** | **4.5** | **2.7** |
| **Guinea** | **Faranah** | **2012** | **92.7** | **67.7** | **86.7** | **64.1** | **20.5** | **43.6** | **53.6** | **81.8** | **59.2** | **25** | **47.6** | **20.4** | **13.1** | **77.1** | **33.1** | **22.7** | **56.8** | **7.9** | **44.1** | **10.2** | **0.7** | **89.2** | **10.8** | **4.5** |
| **Guinea** | **Kankan** | **2012** | **97.7** | **83.2** | **93.6** | **82.7** | **29.9** | **54.6** | **59.2** | **87.2** | **74.6** | **32.3** | **78.9** | **29.8** | **27.3** | **96** | **19.3** | **46.4** | **24.9** | **3.3** | **55.5** | **15.5** | **0.5** | **84** | **16** | **2.8** |
| **Guinea** | **Kindia** | **2012** | **90** | **72.2** | **82.4** | **69.2** | **26.6** | **63.7** | **52.7** | **67.1** | **60.2** | **31.3** | **45.8** | **15.9** | **10** | **87.1** | **28.5** | **56.8** | **86.6** | **23.8** | **27.8** | **8.2** | **3.1** | **88.7** | **11.3** | **2.2** |
| **Guinea** | **Labe’** | **2012** | **94.2** | **67.7** | **77.2** | **56.8** | **26.9** | **75.8** | **49.4** | **80.4** | **63.6** | **33.7** | **43** | **24.5** | **16** | **88.9** | **16.7** | **34.8** | **76.1** | **6.5** | **31** | **6.5** | **0.4** | **93.1** | **6.9** | **2.9** |
| **Guinea** | **Mamou** | **2012** | **95.2** | **82.5** | **90.6** | **80.2** | **32.4** | **81.2** | **43.7** | **67.1** | **61.1** | **33.8** | **72.6** | **38.7** | **30.1** | **83.2** | **32.2** | **58.9** | **68.6** | **6.4** | **35.8** | **7.5** | **0.1** | **92.4** | **7.6** | **2.2** |
| **Guinea** | **N’Ze’re’kore** | **2012** | **91.9** | **77.8** | **83.4** | **76.9** | **32.9** | **55.9** | **60.3** | **73.5** | **58.3** | **34.3** | **61.2** | **30.7** | **27.9** | **85.2** | **32.7** | **48.7** | **55.1** | **16.4** | **54.7** | **9.9** | **0.3** | **89.7** | **10.3** | **6.2** |
| **Guinea** | **Conakry** | **2018** | **96.9** | **78.8** | **82.3** | **72.8** | **35.8** | **65.6** | **65.4** | **79.1** | **66.4** | **41.3** | **53.1** | **55.2** | **36.4** | **NA^b^** | **31.7** | **NA^b^** | **NA^b^** | **NA^b^** | **68.9** | **17.2** | **2.3** | **80.5** | **19.5** | **11** |
| **Guinea** | **Bonke’** | **2018** | **87.8** | **78.3** | **82.8** | **76.3** | **23.3** | **70.3** | **40.7** | **68.6** | **47.7** | **26.4** | **58.8** | **28.9** | **34** | **NA^b^** | **18.1** | **NA^b^** | **NA^b^** | **NA^b^** | **39.2** | **8.8** | **1.7** | **89.5** | **10.5** | **5.9** |
| **Guinea** | **Faranah** | **2018** | **88.5** | **78.6** | **83.7** | **75.7** | **23.1** | **46.3** | **57.5** | **76.9** | **54.4** | **26.1** | **54.9** | **23.9** | **25.6** | **NA^b^** | **31.6** | **NA^b^** | **NA^b^** | **NA^b^** | **34.1** | **7** | **0** | **93** | **7** | **1.9** |
| **Guinea** | **Kankan** | **2018** | **84.3** | **78** | **80.4** | **76.8** | **23.8** | **43.5** | **51.8** | **79.3** | **56.4** | **24.8** | **55.1** | **42.2** | **22** | **NA^b^** | **13.2** | **NA^b^** | **NA^b^** | **NA^b^** | **52.3** | **5.5** | **0.5** | **94** | **6** | **3.1** |
| **Guinea** | **Kindia** | **2018** | **91.9** | **84.2** | **86.3** | **82.1** | **30.7** | **79.6** | **58.4** | **75** | **39.7** | **32.2** | **74.6** | **43.9** | **34.9** | **NA^b^** | **11.1** | **NA^b^** | **NA^b^** | **NA^b^** | **58.5** | **8.8** | **0.9** | **90.3** | **9.7** | **5.9** |
| **Guinea** | **Labe’** | **2018** | **86.3** | **64.1** | **80.2** | **60.6** | **32.2** | **65.9** | **43.4** | **54.5** | **54.8** | **34.6** | **43.2** | **25.3** | **18.7** | **NA^b^** | **39.4** | **NA^b^** | **NA^b^** | **NA^b^** | **46.2** | **6.9** | **0** | **93.1** | **6.9** | **2.2** |
| **Guinea** | **Mamou** | **2018** | **94.4** | **80.2** | **73.5** | **68** | **40.9** | **67.7** | **63.3** | **83.1** | **68.2** | **45.4** | **47.6** | **34.9** | **25.2** | **NA^b^** | **36.6** | **NA^b^** | **NA^b^** | **NA^b^** | **70.2** | **4.9** | **0.4** | **94.7** | **5.3** | **2.7** |
| **Guinea** | **N’Ze’re’kore** | **2018** | **94.2** | **52.6** | **77.2** | **50.5** | **27.1** | **45.7** | **81.5** | **76.7** | **66.2** | **30.5** | **70.3** | **24.1** | **23.6** | **NA^b^** | **14.9** | **NA^b^** | **NA^b^** | **NA^b^** | **41** | **4.8** | **1.7** | **93.4** | **6.6** | **2.5** |
| **Liberia** | **Monrovia** | **2007** | **97.8** | **79.4** | **88.3** | **75.6** | **44** | **82.8** | **66.5** | **80.9** | **76.7** | **52.7** | **56.7** | **26.1** | **20.5** | **76.4** | **70.5** | **65.8** | **72.2** | **28.7** | **51.1** | **7.8** | **0.9** | **91.3** | **8.7** | **3.9** |
| **Liberia** | **North Western** | **2007** | **96.7** | **77.5** | **81.2** | **69.9** | **21.5** | **71** | **39.6** | **72.9** | **69.3** | **27.1** | **72.5** | **14.4** | **12.7** | **44.3** | **59.3** | **65.7** | **90.5** | **18** | **23.6** | **1.7** | **0.1** | **98.3** | **1.7** | **0.4** |
| **Liberia** | **South Central** | **2007** | **97.4** | **66.2** | **70.7** | **58.6** | **23.1** | **71.4** | **46.3** | **69.9** | **67.2** | **35.1** | **65** | **20.9** | **16.7** | **69.6** | **37.9** | **39.5** | **78** | **14** | **28.6** | **6.1** | **0.8** | **93.1** | **6.9** | **1.6** |
| **Liberia** | **South Eastern A** | **2007** | **92.6** | **67.3** | **70.9** | **58.9** | **21.4** | **61.6** | **45.8** | **64.6** | **59** | **28.1** | **67** | **12** | **10.3** | **68.8** | **45.6** | **42.1** | **82** | **20.2** | **27.6** | **3.4** | **0.7** | **95.9** | **4.1** | **1.4** |
| **Liberia** | **South Eastern B** | **2007** | **88.5** | **57.3** | **66** | **51.3** | **17.5** | **54.5** | **39.5** | **62.1** | **51.6** | **23.9** | **56.7** | **12.4** | **11.9** | **72.3** | **38.6** | **37.9** | **73.7** | **17.1** | **15.8** | **2.5** | **0.7** | **96.7** | **3.3** | **1.1** |
| **Liberia** | **North Central** | **2007** | **85.8** | **67.7** | **73** | **64.8** | **30.9** | **61.2** | **47.7** | **62.9** | **56.1** | **34.2** | **53.1** | **11.6** | **7.7** | **66.9** | **50.2** | **40.2** | **76.3** | **21** | **23.7** | **3.1** | **0.5** | **96.4** | **3.6** | **2** |
| **Liberia** | **Monrovia** | **2013** | **99** | **80.6** | **86.1** | **75.5** | **49.6** | **85.3** | **75.9** | **86.6** | **81.4** | **60.6** | **54.1** | **44.5** | **34.7** | **82.1** | **56.4** | **67.6** | **46.8** | **16.3** | **71.4** | **29.4** | **1.7** | **68.9** | **31.1** | **15.9** |
| **Liberia** | **North Western** | **2013** | **98.7** | **87.5** | **87.3** | **82.7** | **38.5** | **74.1** | **57** | **80.7** | **71.1** | **42.4** | **53.7** | **29.5** | **24.5** | **69.4** | **45.4** | **48.2** | **74.8** | **21.3** | **62.4** | **18.1** | **4** | **77.9** | **22.1** | **9.6** |
| **Liberia** | **South Central** | **2013** | **98.8** | **77.1** | **81.4** | **70.7** | **40.9** | **82.8** | **67.2** | **84.4** | **78.5** | **52.2** | **54.2** | **39.5** | **31.3** | **78** | **57** | **63.4** | **48.9** | **16** | **67.8** | **27.6** | **2.2** | **70.2** | **29.8** | **14.8** |
| **Liberia** | **South Eastern A** | **2013** | **94.2** | **78.3** | **79** | **71.5** | **31** | **67.1** | **54.5** | **81.5** | **71.8** | **37.6** | **48.2** | **35.3** | **25.7** | **80.6** | **53.6** | **55.6** | **57.3** | **17.6** | **60** | **22.2** | **4.4** | **73.3** | **26.7** | **11.5** |
| **Liberia** | **South Eastern B** | **2013** | **91.7** | **66** | **68.6** | **58.4** | **22.7** | **65.9** | **43.1** | **71.1** | **59.9** | **29.2** | **51.5** | **31.6** | **26.5** | **77.1** | **42.3** | **44.3** | **52.7** | **8.8** | **48.6** | **14.5** | **4.5** | **80.9** | **19.1** | **7.9** |
| **Liberia** | **North Central** | **2013** | **91.5** | **68.2** | **71.5** | **60.7** | **22.3** | **63.8** | **45.8** | **70** | **67.1** | **29.3** | **49.1** | **26.6** | **19.1** | **65.4** | **35.4** | **31.2** | **67** | **8.9** | **52.7** | **18** | **3.7** | **78.3** | **21.7** | **10** |
| **Liberia** | **Monrovia** | **2019** | **98.9** | **89.7** | **93.8** | **87.3** | **42.3** | **85.6** | **59** | **88.2** | **78.3** | **45.9** | **57.9** | **42.6** | **33.7** | **NA^b^** | **59.1** | **NA^b^** | **NA^b^** | **NA^b^** | **63.7** | **34.8** | **1** | **64.1** | **35.9** | **23.4** |
| **Liberia** | **North Western** | **2019** | **88** | **73.8** | **69.9** | **63.1** | **24.6** | **65.8** | **47.1** | **78** | **62.4** | **31.2** | **52.1** | **34.5** | **29.1** | **NA^b^** | **42.5** | **NA^b^** | **NA^b^** | **NA^b^** | **53.8** | **26.4** | **2** | **71.7** | **28.3** | **17.3** |
| **Liberia** | **South Central** | **2019** | **98.2** | **86.4** | **90.6** | **83.1** | **41.6** | **83.2** | **60** | **86.9** | **77.1** | **46.2** | **56.5** | **43.8** | **33.5** | **NA^b^** | **51.2** | **NA^b^** | **NA^b^** | **NA^b^** | **66** | **34.7** | **1.3** | **64** | **36** | **22.9** |
| **Liberia** | **South Eastern A** | **2019** | **98.3** | **83.9** | **84.2** | **76.5** | **36** | **75** | **61.6** | **79.4** | **71.2** | **42.5** | **55.1** | **41.9** | **35.3** | **NA^b^** | **41.3** | **NA^b^** | **NA^b^** | **NA^b^** | **73** | **34.3** | **4.8** | **60.9** | **39.1** | **17** |
| **Liberia** | **South Eastern B** | **2019** | **93.5** | **82.3** | **81.8** | **75.4** | **32.3** | **71.2** | **59.6** | **71.4** | **67.4** | **37.4** | **70.9** | **43.7** | **39.5** | **NAb** | **39.8** | **NA^b^** | **NA^b^** | **NA^b^** | **60.1** | **17** | **0.9** | **82.1** | **17.9** | **8.6** |
| **Liberia** | **North Central** | **2019** | **94.6** | **78.1** | **81.6** | **70.8** | **26.5** | **70.6** | **45.5** | **78.3** | **64.2** | **32.4** | **60.8** | **32.8** | **27** | **NA^b^** | **32.4** | **NA^b^** | **NA^b^** | **NA^b^** | **66.7** | **31.6** | **1.5** | **66.9** | **33.1** | **22.7** |
| **Sierra Leone** | **Eastern** | **2008** | **88.1** | **62.7** | **66.4** | **55** | **14.2** | **55.9** | **35.2** | **50.1** | **41.5** | **20.7** | **38.8** | **12** | **9.6** | **79.6** | **43** | **49.2** | **69.8** | **17.2** | **26.2** | **3.5** | **1.7** | **94.9** | **5.1** | **1.3** |
| **Sierra Leone** | **Northern** | **2008** | **78.7** | **60.9** | **64.6** | **55.1** | **24.3** | **53.5** | **48.3** | **56.3** | **44.1** | **30.4** | **47.9** | **24** | **19.6** | **61.5** | **34.4** | **47.9** | **61.2** | **13.6** | **21.7** | **3.8** | **1.2** | **95** | **5** | **2.3** |
| **Sierra Leone** | **Southern** | **2008** | **70.9** | **50.3** | **53** | **44.7** | **19.9** | **42.6** | **33.1** | **48.5** | **43.3** | **23** | **49.9** | **17.8** | **16.4** | **78.6** | **36.5** | **46.1** | **52** | **12.3** | **28.2** | **5.3** | **1.2** | **93.5** | **6.5** | **3.3** |
| **Sierra Leone** | **Western** | **2008** | **97.6** | **75.7** | **83.3** | **70.1** | **39.9** | **77.4** | **65** | **79.5** | **67.2** | **49.3** | **64.3** | **40.4** | **32.4** | **79.4** | **49.1** | **68.1** | **52.9** | **15.7** | **62.2** | **17.5** | **1.3** | **81.2** | **18.8** | **7.5** |
| **Sierra Leone** | **Eastern** | **2013** | **95.7** | **74.2** | **77.4** | **68.8** | **20.4** | **58.9** | **47.6** | **54.3** | **52.7** | **22.2** | **51.5** | **43** | **29.8** | **89.5** | **29.4** | **43.2** | **52.1** | **6.6** | **67.2** | **13** | **3** | **84.1** | **15.9** | **4.3** |
| **Sierra Leone** | **Northern** | **2013** | **95.7** | **77.4** | **81.9** | **73.1** | **31.4** | **64.7** | **57.6** | **56.6** | **62.7** | **34.7** | **43.5** | **42.1** | **29.5** | **77.7** | **43.6** | **53.1** | **29.7** | **2.6** | **65.1** | **10.8** | **3.8** | **85.4** | **14.6** | **5.4** |
| **Sierra Leone** | **Southern** | **2013** | **98.2** | **86.1** | **88.2** | **81.4** | **40.4** | **81.1** | **64** | **80** | **62.9** | **44.2** | **52.6** | **49.3** | **36.7** | **87.8** | **53.5** | **64.8** | **36.8** | **13.2** | **85.5** | **22.8** | **3.4** | **73.8** | **26.2** | **9.4** |
| **Sierra Leone** | **Eastern** | **2019** | **97.9** | **88.6** | **91.3** | **87.1** | **44.4** | **72.6** | **73.5** | **76.7** | **61.2** | **46.6** | **67.4** | **33.3** | **26.6** | **NA^b^** | **34.6** | **NA^b^** | **NA^b^** | **NA^b^** | **82.7** | **19.1** | **3** | **77.9** | **22.1** | **8.8** |
| **Sierra Leone** | **Northern** | **2019** | **94.5** | **85.5** | **88.3** | **82.4** | **31.3** | **63.7** | **69.8** | **74.4** | **47.7** | **32.3** | **67.6** | **37.1** | **35** | **NA^b^** | **24.3** | **NA^b^** | **NA^b^** | **NA^b^** | **69.1** | **19.8** | **2.4** | **77.7** | **22.3** | **12.8** |
| **Sierra Leone** | **Southern** | **2019** | **93** | **81.7** | **82.3** | **75.9** | **28.9** | **68.3** | **63.7** | **74.1** | **43.6** | **33.2** | **69.6** | **38.1** | **32.9** | **NA^b^** | **26.8** | **NA^b^** | **NA^b^** | **NA^b^** | **61.6** | **15.2** | **2.3** | **82.6** | **17.4** | **9.5** |
| **Sierra Leone** | **Western** | **2019** | **97.3** | **83.6** | **85.5** | **78.5** | **36.3** | **76.4** | **68.8** | **75.9** | **55.7** | **39.3** | **78.9** | **45.4** | **38.6** | **NA^b^** | **38.1** | **NA^b^** | **NA^b^** | **NA^b^** | **71.6** | **27.7** | **4** | **68.3** | **31.7** | **16.5** |
| **Sierra Leone** | **North Western** | **2019** | **95.9** | **87.5** | **86.7** | **80.7** | **21.6** | **63.3** | **50** | **74.9** | **44.7** | **24.9** | **70.5** | **50.4** | **48.5** | NA^b^ | **20.7** | NA^b^ | NA^b^ | NA**^b^** | **67.8** | **15.5** | **1.4** | **83.1** | **16.9** | **10.3** |

**^a^ICF, 2015. The DHS Program STATcompiler. Funded by USAID. http://www.statcompiler.com. December 29 2021**

**^b^NA = Not applicable; MTCT = Mother to child transmission**

**CCK1 =Men who have heard of HIV or AIDS**

**CCK2 =Knowledge of HIV prevention methods- use of condom**

**CCK3 =Knowledge of HIV prevention methods- one partner only**

**CCK4 =Knowledge of HIV prevention methods composite of 2 components**

**CCK5 =Comprehensive correct knowledge about AIDS**

**CCK6=No incorrect beliefs about AIDS- a healthy looking person can have the AIDS virus**

**CCK7 =No incorrect beliefs about AIDS- AIDS cannot be transmitted by mosquito bites**

**CCK8=No incorrect beliefs about AIDS- AIDS cannot be transmitted by supernatural means**

**CCK9 =No incorrect beliefs about AIDS- cannot become infected by sharing food with someone who has AIDS**

**CCK10 =No incorrect beliefs about AIDS- composite of 4 components**

**CCK11 =Knowledge of MTCT- Through breast feeding**

**CCK12 =prevention Knowledge of MTCT- can be prevented by mother taking special drugs during pregnancy**

**CCK13 =Knowledge of prevention of mother to child transmission of HIV**

**CCK14 =Accepting attitudes -willing to care for family member sick with AIDS**

**CCK15 =Accepting attitudes -would buy fresh vegetables from a shopkeeper with AIDS**

**CCK16 =Accepting attitudes – Female teacher who is HIV+ but not sick should be allowed to continue teaching in school**

**CCK17 =Accepting attitudes – Not secretive about family member's HIV status**

**CCK18 =Accepting attitudes towards those living with HIV- composites of 4 components**

**T1 =Men who know where to get an HIV test**

**T2 =Men ever tested for HIV and received test results**

**T3 =Men ever tested for HIV and did not receive test results**

**T4 =Men never tested for HIV**

**T5 =Men ever receiving an HIV test**

**T6 =Men receiving an HIV test and receiving test results in the last 12 months before survey.**
